# The dinosaur tracks of Tyrants Aisle: An Upper Cretaceous ichnofauna from Unit 4 of the Wapiti Formation (upper Campanian), Alberta, Canada

**DOI:** 10.1371/journal.pone.0262824

**Published:** 2022-02-02

**Authors:** Nathan J. Enriquez, Nicolás E. Campione, Matt A. White, Federico Fanti, Robin L. Sissons, Corwin Sullivan, Matthew J. Vavrek, Phil R. Bell

**Affiliations:** 1 Palaeoscience Research Centre, School of Environmental and Rural Science, University of New England, Armidale, NSW, Australia; 2 Dipartimento di Scienze della Terra e Geologico-Ambientali, Alma Mater Studiorum, Università di Bologna, Bologna, Italy; 3 Department of Biological Sciences, University of Alberta, Edmonton, Alberta, Canada; 4 Philip J. Currie Dinosaur Museum, Wembley, Alberta, Canada; 5 Cutbank Palaeontological Consulting, Grande Prairie, Alberta, Canada; 6 Department of Natural History, Royal Ontario Museum, Toronto, Ontario, Canada; Southern Methodist University, UNITED STATES

## Abstract

The Wapiti Formation of northwest Alberta and northeast British Columbia, Canada, preserves an Upper Cretaceous terrestrial vertebrate fauna that is latitudinally situated between those documented further north in Alaska and those from southern Alberta and the contiguous U.S.A. Therefore, the Wapiti Formation is important for identifying broad patterns in vertebrate ecology, diversity, and distribution across Laramidia during the latest Cretaceous. Tracksites are especially useful as they provide a range of palaeoecological, palaeoenvironmental, and behavioural data that are complementary to the skeletal record. Here, we describe the Tyrants Aisle locality, the largest *in-situ* tracksite known from the Wapiti Formation. The site occurs in the lower part of Unit 4 of the formation (~72.5 Ma, upper Campanian), exposed along the southern bank of the Redwillow River. More than 100 tracks are documented across at least three distinct track-bearing layers, which were deposited on an alluvial floodplain. Hadrosaurid tracks are most abundant, and are referable to *Hadrosauropodus* based on track width exceeding track length, broad digits, and rounded or bilobed heel margins. We suggest the hadrosaurid trackmaker was *Edmontosaurus regalis* based on stratigraphic context. Tyrannosaurids, probable troodontids, possible ornithomimids, and possible azhdarchid pterosaurs represent minor but notable elements of the ichnofauna, as the latter is unknown from skeletal remains within the Wapiti Formation, and all others are poorly represented. Possible social behaviour is inferred for some of the hadrosaurid and small theropod-like trackmakers based on trackway alignment, suitable spacing and consistent preservation. On a broad taxonomic level (i.e., family or above), ichnofaunal compositions indicate that hadrosaurids were palaeoecologically dominant across Laramidia during the late Campanian within both high-and low-latitude deposits, although the role of depositional environment requires further testing.

## Introduction

Tracks provide valuable data on behavioural, palaeoenvironmental, and palaeoecological aspects of dinosaur biology [1]. Such insights complement the skeletal record and allow better exploration of hypotheses that are difficult to test using body fossils alone, such as those pertaining to herding behaviours [2], palaeoenvironmental preferences [1, 3, 4], and dinosaur speeds and gaits [5–7]. However, isolated and *ex-situ* tracks provide fewer data than multi-taxic, *in-situ* track assemblages. Specifically, isolated tracks lack information about the speed or gait of their trackmakers, and cannot be used to infer social behaviour, which requires observation of multiple footfalls and their spatial relationships among any other tracks on the same bedding plane. *Ex-situ* tracks also lack precise stratigraphic context, making it difficult to assess their contextual relevance to other *in-situ* fossil-bearing layers. Therefore, the utility of tracks in studies of palaeoecology and extinct animal behaviour is limited by the nature of their preservation and physical exposure.

The vertebrate track record of the Wapiti Formation, which crops out in NW Alberta and NE British Columbia, largely consists of isolated, *ex-situ* prints that occur on fallen blocks found on modern river margins [8–11]. Indeed, the vast majority of vertebrate fossils from the Wapiti Formation are recovered as isolated remains from alongside rivers, where cliff and bank exposures provide the best access to the fossil-bearing strata. Nevertheless, a rich vertebrate fossil record from the formation is emerging, and already includes skeletal remains of a variety of theropod, hadrosaurid, ceratopsian, ankylosaurian, and possible thescelosaurid dinosaurs [8, 10, 12–16]. Excluding the locality described herein, only a single large *in-situ* tracksite has previously been described from the formation, which preserves footprints attributed to *Hadrosauropodus*, *Bellatoripes fredlundi* and *Saurexallopus cordata*, near Tumbler Ridge in British Columbia [11, 17].

Fossil faunas from the Wapiti Formation are significant as they bridge the latitudinal gap between partly coeval faunas from the contiguous U.S.A. (e.g., those of the Two Medicine and Judith River formations [18–22]) and southern Alberta (e.g., within the Dinosaur Park and Horseshoe Canyon formations [23–25]) with those further north in Alaska (e.g., within the Prince Creek, Cantwell, and Chignik formations [26–32]). Therefore, sampling of Wapiti Formation faunas contributes to a more complete picture of latitudinal variation in dinosaur diversity, evolution, and biogeography within Laramidia. In addition, the Wapiti Formation represents a near continuous terrestrial record during portions of the Campanian where marine transgressive deposits of the Bearpaw Sea interrupt the terrestrial fossil record in places such as southern Alberta and Montana [12, 33, 34]. Thus, the Wapiti strata are an important window into Laramidian terrestrial vertebrate evolution and diversity during temporal intervals that are poorly represented elsewhere.

**Fig 1:**
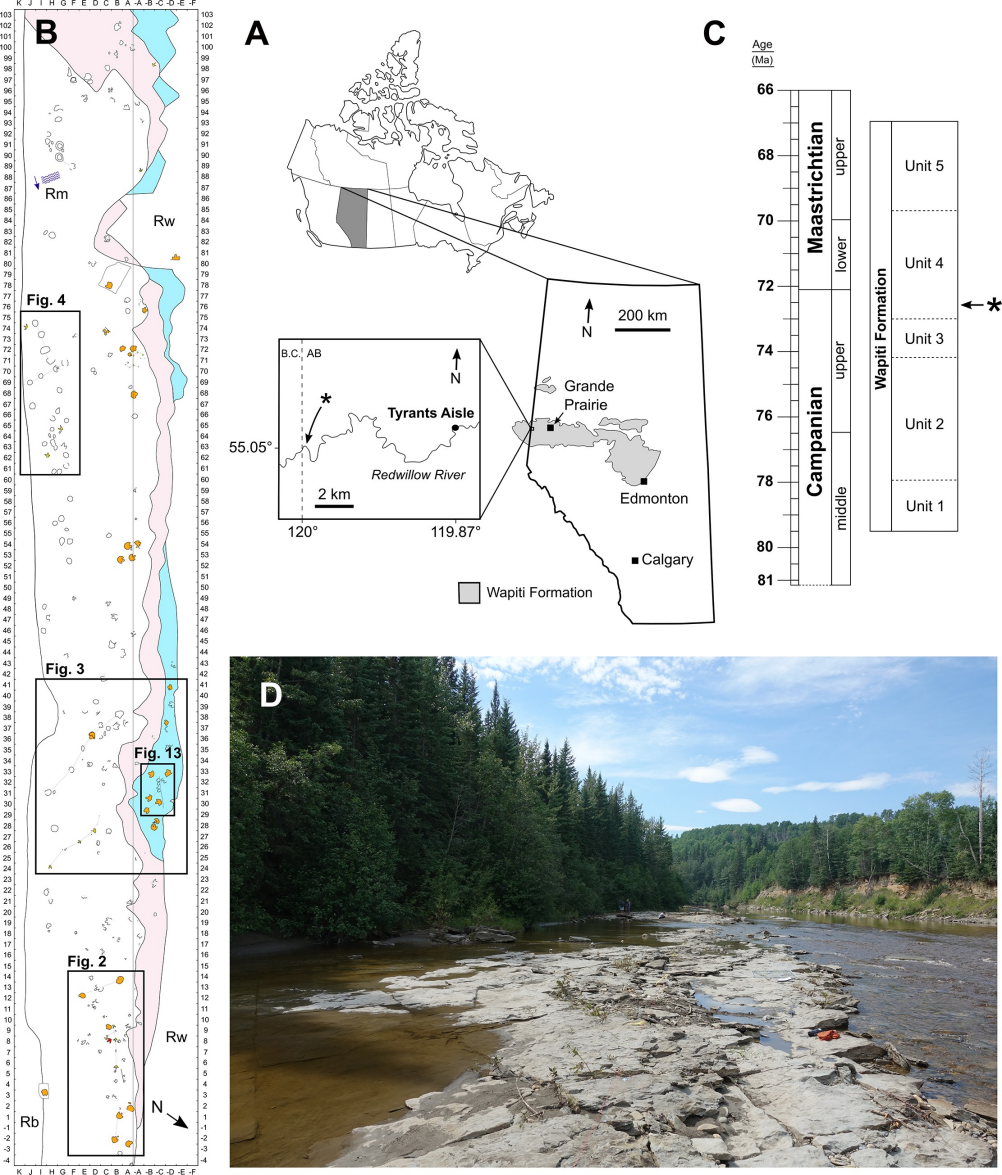
**A**, Location of the Tyrants Aisle dinosaur tracksite in western Alberta, Canada. Light grey shading denotes the extent of the Wapiti Formation, retraced and modified from [12]. The asterisk (*) denotes the location of a roughly coeval (or slightly stratigraphically lower) bentonite layer dated to 72.58±0.09 Ma using Ar/Ar dating [15]. Map of Canada and Alberta retraced and modified from [38]. **B**, Grid map overview of the entire tracksite. Colours identify stratigraphic layers, as follows: light blue = track layer 1; pink = track layer 2; white = track layer 3. Increments on *x-*and *y-*axes mark one metre intervals. Ripple marks (in dark blue; with localised current direction indicated) are not to scale. **C**, Stratigraphic position (*) within Unit 4 of the Wapiti Formation of the aforementioned bentonite layer, which provides an approximate maximum age for the Tyrants Aisle tracksite. Unit ages based on [39]. **D**, Photograph of Tyrants Aisle in 2018 during a period of low water level, looking west-southwest. Abbreviations: AB = Alberta; B.C. = British Columbia; N = north; Rb = riverbank; Rm = ripple marks; Rw = Redwillow River.

A field party from the Royal Tyrrell Museum of Palaeontology (TMP) discovered the first large *in-situ* dinosaur tracksite from the Wapiti Formation in 1989, ~66 km WSW of the city of Grande Prairie, in northwestern Alberta (Fig 1A). Approximately a dozen hadrosaurid tracks—originally referred to *Amblydactylus—*were initially identified (‘Red Willow River hadrosaur ichnites site #1’ in Tanke [8]). After an additional brief visit by the TMP in 2003, it was noted that the site “requires some salvage efforts and research attention before the prints are lost forever” [8 p. 9] owing to its position within the channel of the modern-day Redwillow River and high seasonal discharge. More recently, a collection of theropod footprints identified at the locality—renamed Tyrants Aisle—were studied by Enriquez et al. [35–37], although the majority of the tracksite remains undescribed. Therefore, the purpose of this paper is to provide the first detailed description and palaeobiological synthesis of the whole locality, which remains the largest *in-situ* tracksite known from the Wapiti Formation to date. Crucially, its large size and *in-situ* preservation provides the broadest available ichnological perspective on the palaeoecology of dinosaurs within the Wapiti Formation, and the strongest basis for assessments of ichnofaunal diversity, relative abundance and trackmaker behaviour.

**Institutional abbreviations**—**PRPRC**, Peace Region Palaeontology Research Centre, Tumbler Ridge, British Columbia, Canada; **ROM**, Royal Ontario Museum, Toronto, Ontario, Canada; **TMP**, Royal Tyrrell Museum of Palaeontology, Drumheller, Alberta, Canada; **UALVP**, University of Alberta Laboratory for Vertebrate Palaeontology, Edmonton, Alberta, Canada.

## Geographic and geological setting

### Study area

The Tyrants Aisle locality is exposed ~66 kilometres WSW of Grande Prairie as a series of rock ledges within the active channel of the Redwillow River (precise GPS coordinates on file at the UALVP) (Fig 1A). The tracks documented herein occur on at least three discrete stratigraphic layers distributed across a continuous area more than 105 metres in length and 7.5–15 metres in width (Fig 1B). Erosion by the river, and visitors traversing the site on ATVs (all-terrain vehicles), have caused degradation of the track-bearing surfaces [8].

#### Regional stratigraphic context and age

The track-bearing horizons at Tyrants Aisle belong to Unit 4 of the Wapiti Formation, which is a coal-rich unit containing channel-fill, overbank, levee and crevasse splay deposits [33, 40]. Unit 4 dates from the late Campanian to earliest late Maastrichtian [10, 33, 39]. Tyrants Aisle is positioned within lower Unit 4 (Fig 1C)—and based on observations of lateral continuity—occurs at approximately the same stratigraphic level as (or slightly above) a bentonite layer situated ~16 km upstream near Red Willow Falls, which has been Ar/Ar dated to 72.58±0.09 Ma [15]. Thus, the tracksite can be confidently regarded as late Campanian in age. In its entirety, Unit 4 is laterally equivalent to at least the Drumheller, Horsethief and Morrin members of the Horseshoe Canyon Formation within southern Alberta, and possibly also the Tolman Member [34, 39, 41, 42]. Within lower Unit 4, Tyrants Aisle is specifically correlated with the Drumheller Member, and with the *Edmontosaurus regalis-Pachyrhinosaurus canadensis* dinosaur biozone of the Horseshoe Canyon Formation [24, 34, 39, 42].

## Materials and methods

### Site documentation, track measurements and numbering

Tyrants Aisle is only exposed for a few weeks each year, typically in summer and/or autumn when water levels are sufficiently low and the landscape is not yet snow-covered. Fieldwork associated with this study was conducted under a permit to excavate palaeontological resources issued by the government of Alberta to C. S. (permit number 18–033). Most data were collected in August during the 2018 Boreal Alberta Dinosaur Project (BADP) fieldwork season. Attempts to access the site in 2017 and 2019 were thwarted by high water levels.

**Fig 2:**
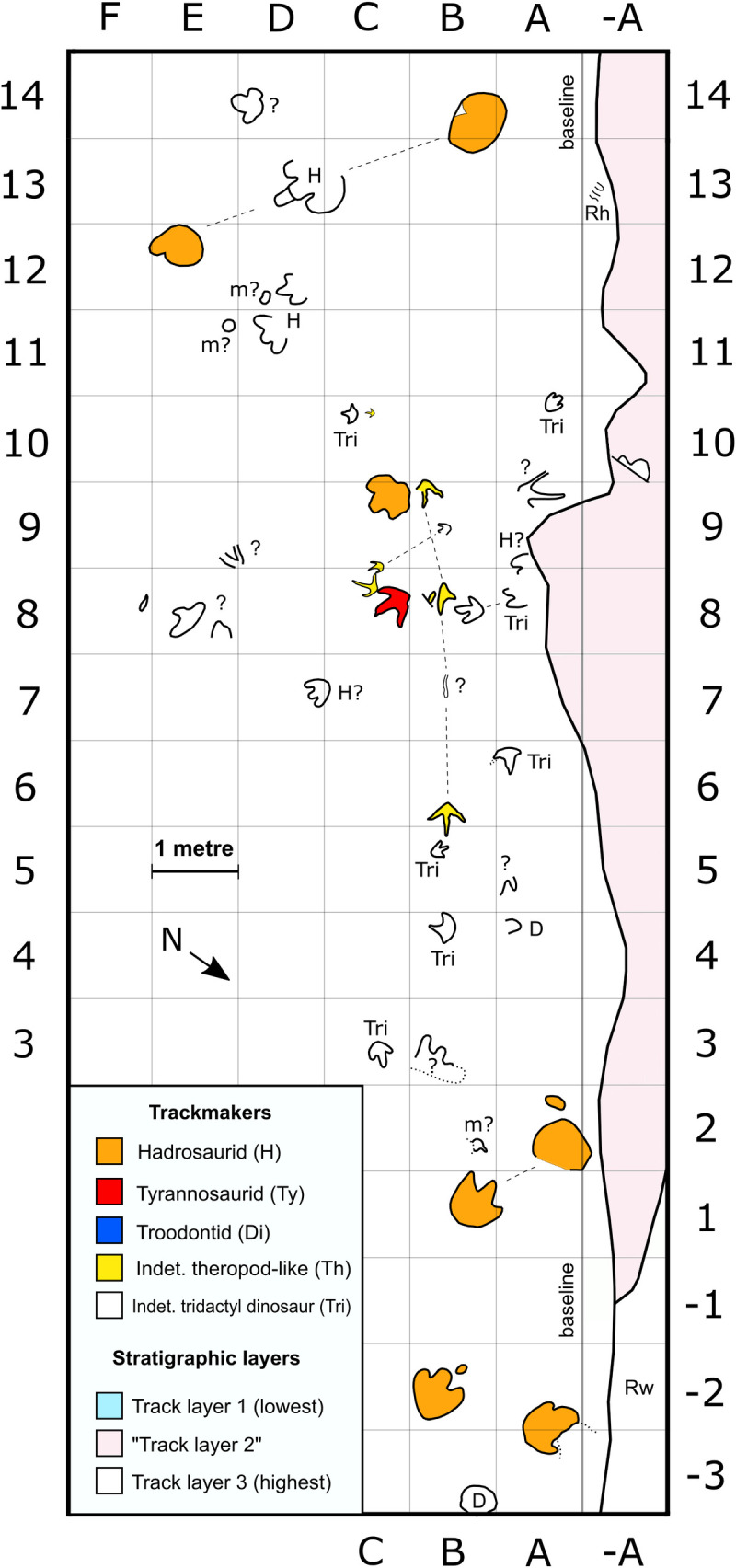
Enlargement of tracksite area containing tyrannosaurid, other theropod-like, and hadrosaurid tracks. Each grid square is 1 m^2^. Dashed lines associate tracks within trackway sequences, while dotted lines indicate eroded or uncertain track features. Trackmakers are indicated using colour fills for enclosed footprints, or abbreviations for most open track outlines. Question marks indicate ambiguous traces. Quotation marks around “track layer 2” reflect the likely presence of more than one distinct layer within this stratigraphic interval. Abbreviations: D = depression; H = hadrosaurid; m = manus impression; Rh = *Rhizocorallium* traces (not to scale); Rw = Redwillow River; Th = indet. theropod-like track; Tri = indet. tridactyl dinosaur; Ty = tyrannosaurid. As the legend shown here is continuous across Figs 2–4, not all trackmakers or stratigraphic layers depicted in the key are present within this figure.

**Fig 3:**
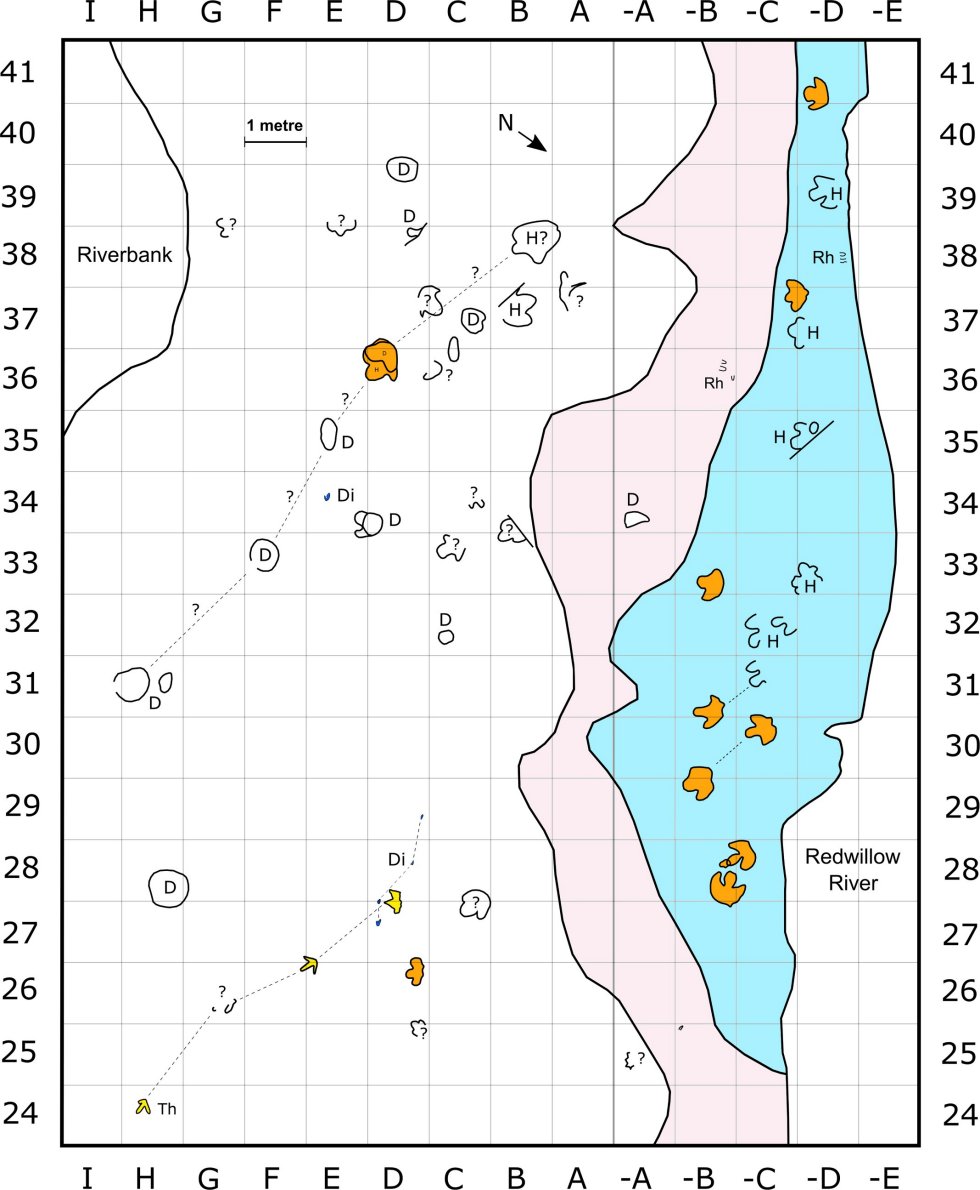
Enlargement of tracksite area containing probable deinonychosaur, other theropod-like, and hadrosaurid tracks on track layer 3 (white), and parallel hadrosaurid tracks on track layer 1 (light blue). Track type and stratigraphic layer colours follow Fig 2. Each grid square is 1 m^2^. Dashed lines associate tracks within trackway sequences. Trackmakers are indicated using colour fills for enclosed footprints, or abbreviations for most open track outlines. Question marks indicate ambiguous traces or trackway associations. Abbreviations: Di = probable deinonychosaur; H = hadrosaurid; Th = indet. theropod-like track; Rh = *Rhizocorallium* traces (not to scale); D = depression.

**Fig 4:**
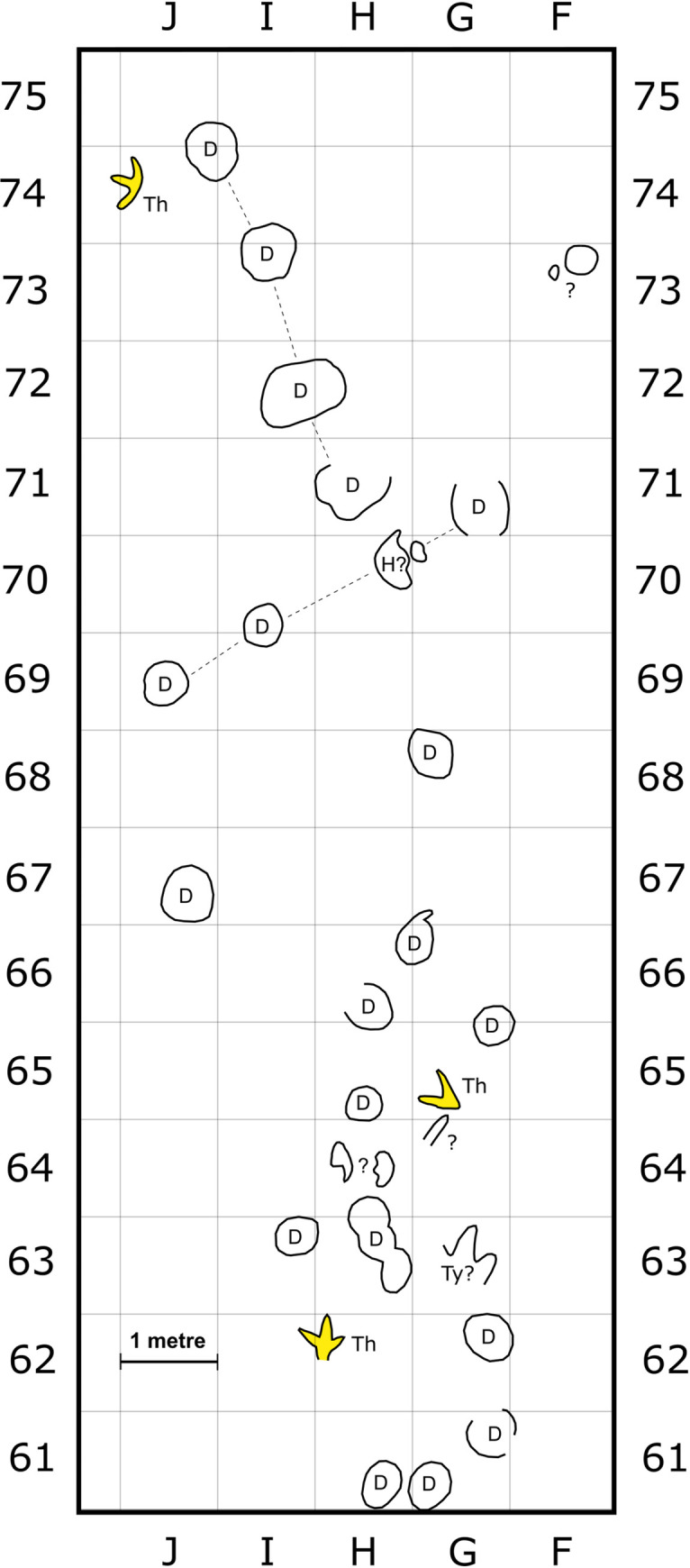
Enlargement of tracksite area on track layer 3, containing a high concentration of eroded footprints. Track type colours follow Fig 2. Each grid square is 1 m^2^. Dashed lines associate tracks within trackway sequences. Trackmakers are indicated using colour fills for enclosed footprints, or abbreviations for most open track outlines. Question marks indicate ambiguous traces. Abbreviations: D = depression; H = hadrosaurid; Th = indet. theropod-like track; Ty = tyrannosaurid.

The site was hand mapped using a baseline and a portable 1x1 m grid square. The hand-drawn maps were digitised and combined into a single comprehensive site map created with Inkscape v. 0.92 (Figs 1B and 2–4). In total, the mapped area measured ~1, 400 m^2^. Measurements of the best-preserved and most distinct tracks of each observable morphotype were collected on-site using a tape measure, and additional measurements were collected digitally in Inkscape from either a scaled photograph, map, or digital elevation model.

**Fig 5:**
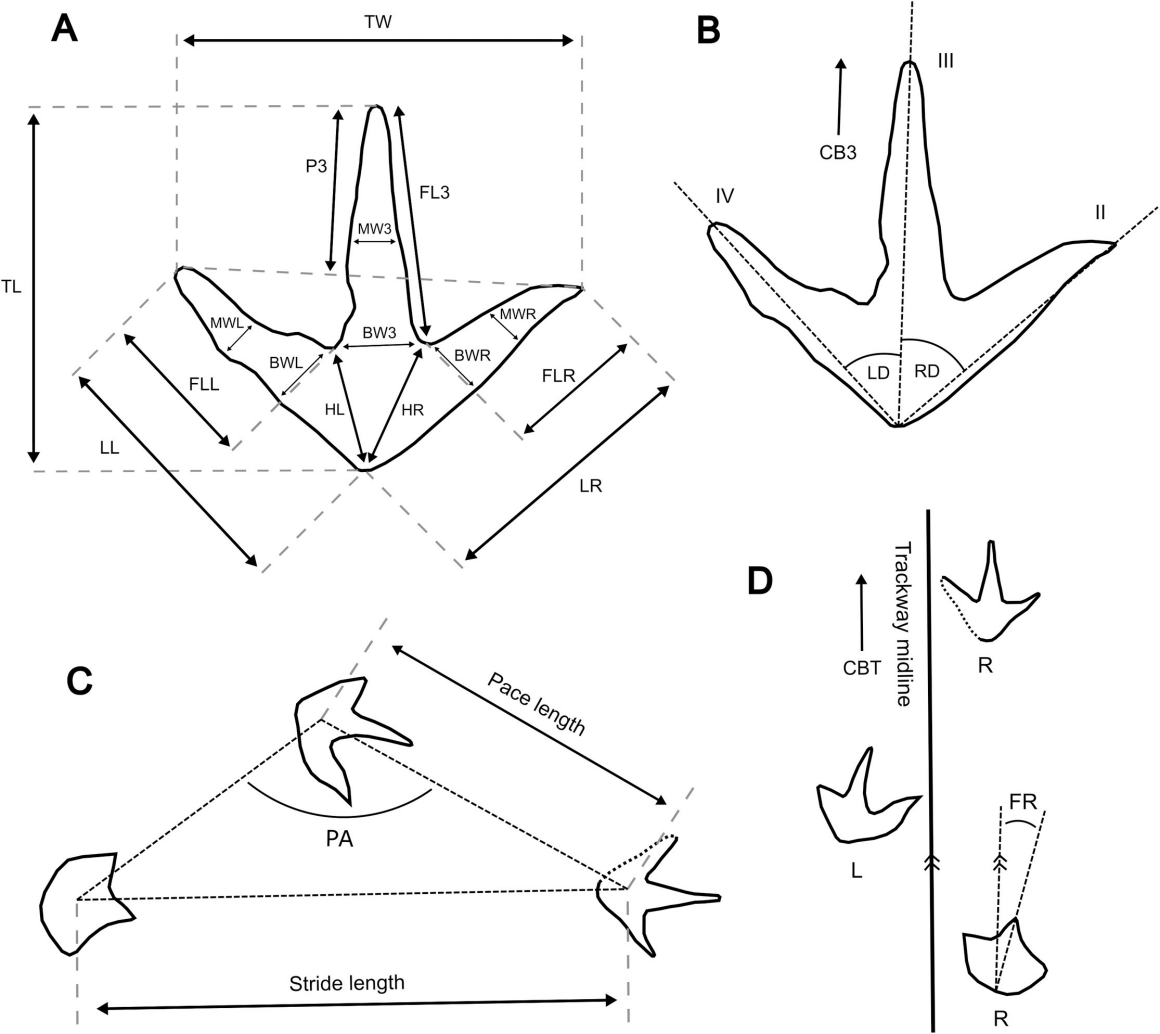
Measurements used in this study to characterise tridactyl tracks and trackways. Where shown, L = left track; R = right track. As many tracks could not be confidently attributed to either the left or right foot, certain measurements are given in terms of their position relative to digit III. **A**, Linear measurements of individual tracks (all in cm): TL = track length; TW = track width; LL = heel to leftmost digit tip; LR = heel to rightmost digit tip; FLL = free length of the leftmost digit; FL3 = free length of digit III; FLR = free length of the rightmost digit; P3 = projection of digit III beyond digits II and IV; HL = heel to leftmost digit hypex; HR = heel to rightmost digit hypex; BWL = basal width of the leftmost digit; BW3 = basal width of digit III; BWR = basal width of the rightmost digit, MWL = midpoint width of the leftmost digit, MW3 = midpoint width of digit III; and MWR = midpoint width of the rightmost digit (adapted from [43]). **B**, Angular measurements of individual tracks: CB3 = 360° compass bearing of the digit III midline (east from north); LD = divarication angle between digit III and the leftmost digit (in plan view); RD = divarication angle between digit III and the rightmost digit (in plan view). **C**–**D**, Trackway measurements: PA = pace angulation; CBT = 360° compass bearing of the trackway midline (east from north); FR = foot rotation; stride and pace length measurements are also shown.

Measurements largely followed Therrien et al. [43] and Salisbury et al. [44] and, for individual footprints, included: compass bearing, track length, track width, basal digit lengths, heel to digit tip lengths, heel to hypex lengths, divarication angles, length of digit III extension beyond the tips of II and IV, widths of digit bases, and widths of digits at their mid-points (Fig 5A and 5B). For trackway sequences—defined here as two or more sequential tracks produced by the same individual—additional measurements included (where possible) pace and stride lengths, pace angulation, relative foot rotation, and compass bearing of the trackway midline (Fig 5C and 5D).

Representative tracks were moulded with Dragon Skin silicone (Smooth-On, Inc) and are accessioned at the UALVP. The moulded footprints were a large theropod track (Th.Tw1.4.6B; UALVP 59918) and adjacent indeterminate tridactyl print (Tri.I.5B; UALVP 59919) on the same piece of silicone, as well as a hadrosaurid pes print (H.I.28-B; UALVP 59917), and a probable small theropod trackway comprising three consecutive prints (Th.Tw5.71-A–72-A; UALVP 59920).

Oriented rock samples from each track layer were also collected for thin sectioning and petrographic observation. Samples were ground down and set on top of glass slides at the University of New England in Armidale, Australia. Viewing and imaging of each slide was performed at Macquarie University in Sydney, Australia with a Nikon Eclipse 50iPOL petrographic microscope, equipped with a Nikon DS-Fi1 digital camera, and using Nikon NIS Elements-D software to manipulate image settings.

Throughout this work, individual tracks or trackways are documented using a numbering system that allows specific specimens to be referred to in-text. When referring to an isolated track, or a trackway sequence as a whole, the system uses a three-part code, which first broadly identifies the trackmaker type (H = hadrosaurid; Ty = tyrannosaurid; Di = didactyl theropod; Th = indeterminate theropod-like trackmaker; Tri = indeterminate tridactyl dinosaur; In = indeterminate trace), followed by an indication of whether the code refers to an isolated track or a trackway (I = isolated; Tw = trackway) and finally the grid location of the specimen within the overall tracksite (e.g. 8B or 32-C, the hyphen in the latter indicating that the grid is on the negative side of the baseline). For tracks that were identified outside the main study area, the location is designated simply as OG (outside gridded area). When a single track was spread across multiple grids, the grid that contained the largest portion of the track was taken as its location. For trackways, which almost always span multiple grids, the grid locations of the first and last footfalls were given as a range. Trackways were also numbered in order of their proximity to the beginning of the baseline, to distinguish between different trackways produced by the same type of trackmaker. For example, the code Th.Tw1.9B–6B pertains to the first theropod-like trackway (i.e., closest to the beginning of the baseline), whose first and final prints are located in grids 9B and 6B, respectively, while print H.I.28-B is an isolated hadrosaurid track located in grid 28-B. When referring to specific tracks within a trackway sequence, a four-part code is used, identifying the trackmaker first, followed by the trackway number, the number of the print within the sequence, and finally the grid location of the print. For example, Th.Tw3.4.24H is the fourth footprint in the third theropod-like trackway, and is located in grid 24H. The utility of these codes is that they allow any track referred to in-text to be located on the corresponding tracksite maps (Figs 1B and 2–4).

### Photogrammetry and interpretive outlines

Most tracks were photographed using a Nikon D810 SLR camera and 24 mm lens. For the best-preserved examples, these images were used to reconstruct photogrammetric 3D digital elevation models (DEMs), from which final interpretive track outlines were produced. All digital models used within this study—and the photographs used to create them—are available for download within the supplementary information, in accordance with suggested practice [45, 46]. To build the DEMs, images were first imported into VisualSFM v.0.5.26 [47] to produce sparse point cloud reconstructions. Dense point cloud reconstructions were created using CMVS/PMVS [48, 49] and then trimmed in MeshLab version 2016.12 [50, 51], following which a Poisson surface reconstruction was performed. DEMs of the Poisson meshes were produced using Cloud Compare v.2.9.1 [52] and final colour edits made in Paraview v.5.5.2 [53]. Outline interpretations of tracks were then drawn in Inkscape v.0.92 using the DEMs. These outlines, together with the DEMs, silicone moulds, on-site photographs, and track measurements, formed the basis for our descriptions of the track morphotypes.

### Identification of trackmaker morphotypes

All tracks were identified to the lowest possible taxonomic level, using their overall morphological characters and contextual (i.e., chronostratigraphic) relevance. Based on skeletal material recovered within Late Cretaceous terrestrial strata of both the Wapiti Formation and more broadly in western Canada [8, 12, 15, 16, 54, 55], tridactyl prints from Tyrants Aisle can be confidently regarded as belonging to either hadrosaurid, theropod or, possibly, thescelosaurid dinosaurs; tracks that possess broad, rounded digits with blunt terminations and relatively broad heels were treated as hadrosaurid tracks, while prints with relatively slender digits, sharp claw marks, and more narrow heels were regarded as theropod-like tracks [7, 56]. The term “theropod-like” is used herein to accommodate the theoretical possibility that some of these tracks may pertain to thescelosaurids, which arguably produced similar track morphologies to those of theropods, making them difficult to distinguish. While thescelosaurids are presently unknown within Unit 4 of the Wapiti Formation, possible fragmentary remains of these dinosaurs have been recovered from Unit 3 [12].

Tridactyl theropod-like tracks were sorted into tyrannosaurid or indeterminate theropod-like morphotypes, primarily according to size. As tyrannosauroids are the only known large-bodied functionally-tridactyl theropods within post-Cenomanian strata from North America [57, 58], and given the presence of indeterminate tyrannosaurid body fossils and tracks within the Wapiti Formation [10, 12, 14, 17], it was assumed that any tridactyl theropod-like track ≥45 cm in length—that does not show evidence of being a deep undertrack [59]—must belong to a tyrannosaurid maker. Deep undertracks may be considerably larger than the corresponding surface track and shallow undertracks, but can be recognised by their poorly defined, flattened track margins, and by the absence of such features as sharp claw marks, digital pad impressions, and skin impressions [60–64]. Our threshold value of 45 cm is based on the largest plausible foot length for non-tyrannosaurid tridactyl theropods within post-Cenomanian strata from western Canada. This value was found by Enriquez et al. [35] to be approximately 40 cm based on the largest known, fragmentary ornithomimid pedal unguals recovered from the mid–late Campanian Dinosaur Park Formation in southern Alberta [65], and scaling these with reference to complete ornithomimid pedes [66, 67]. Thus, below 45 cm, we acknowledge that there is potentially size overlap between multiple major theropod clades and Thescelosauridae, and refer most of these tracks to indeterminate theropod-like trackmakers. However, some examples are tentatively identified as possible ornithomimid tracks (see ‘taxonomic affinities’). Non-tyrannosaurid theropod-like tracks are here divided arbitrarily into small (track length < 25 cm), medium (25–35 cm), and large (>35–45 cm) indeterminate size classes, although there is some morphological overlap. Thus, each size class does not necessarily pertain to a distinct trackmaker. In addition to the tridactyl footprints, a collection of small, didactyl tracks from Tyrants Aisle are probably those of troodontid theropods [37].

### Trackmaker hip height and speed estimation

Estimation of hip height for non-tyrannosaurid theropod-like trackmakers and facultatively bipedal hadrosaurids followed the general rule first applied by Alexander [5] and supported by Henderson [68], namely that hip height is approximately four times the length of the print.

For tyrannosaurid trackmakers, hip height estimation follows equation 4 from McCrea et al. [17], a tailored formula derived from hind limb and phalangeal measurements of *Albertosaurus*, *Gorgosaurus*, and *Daspletosaurus*. To account for bending at the knee and ankle, modification of the original equation is needed, where the estimated hip height (*h*) is multiplied by 0.8. Both *h* and foot length (*FL*) are in millimetres.


$$h\approx \left(29.8\;x\;FL^{0.711}\right)\;x\;0.8$$

For track sequences with observable stride length, trackway maker speed was estimated according to Alexander’s [5] equation for bipedal dinosaurs:

$$V\approx 0.25\;x\;g^{0.5}\;x\;\lambda^{1.67}\;x\;h^{‐1.17}$$

Where *V* is the velocity in metres per second, g is the acceleration due to gravity (approximately equal to 9.8 m/s^2^), λ is the stride length in metres, and *h* is the hip height, also in metres.

## Depositional environment, tracksite stratigraphy and lithology

Sediments from Unit 4 of the Wapiti Formation, including those at Tyrants Aisle, accumulated on floodplains situated between the newly forming Rocky Mountains to the west and the receding Western Interior Seaway further east [33, 40]. While Tyrants Aisle is currently situated at 55.064°N, its palaeolatitude within Laramidia during the late Campanian is estimated to have been ~63.5°N [69, 70]. Therefore, the site records a relatively high latitude dinosaur assemblage.

**Fig 6:**
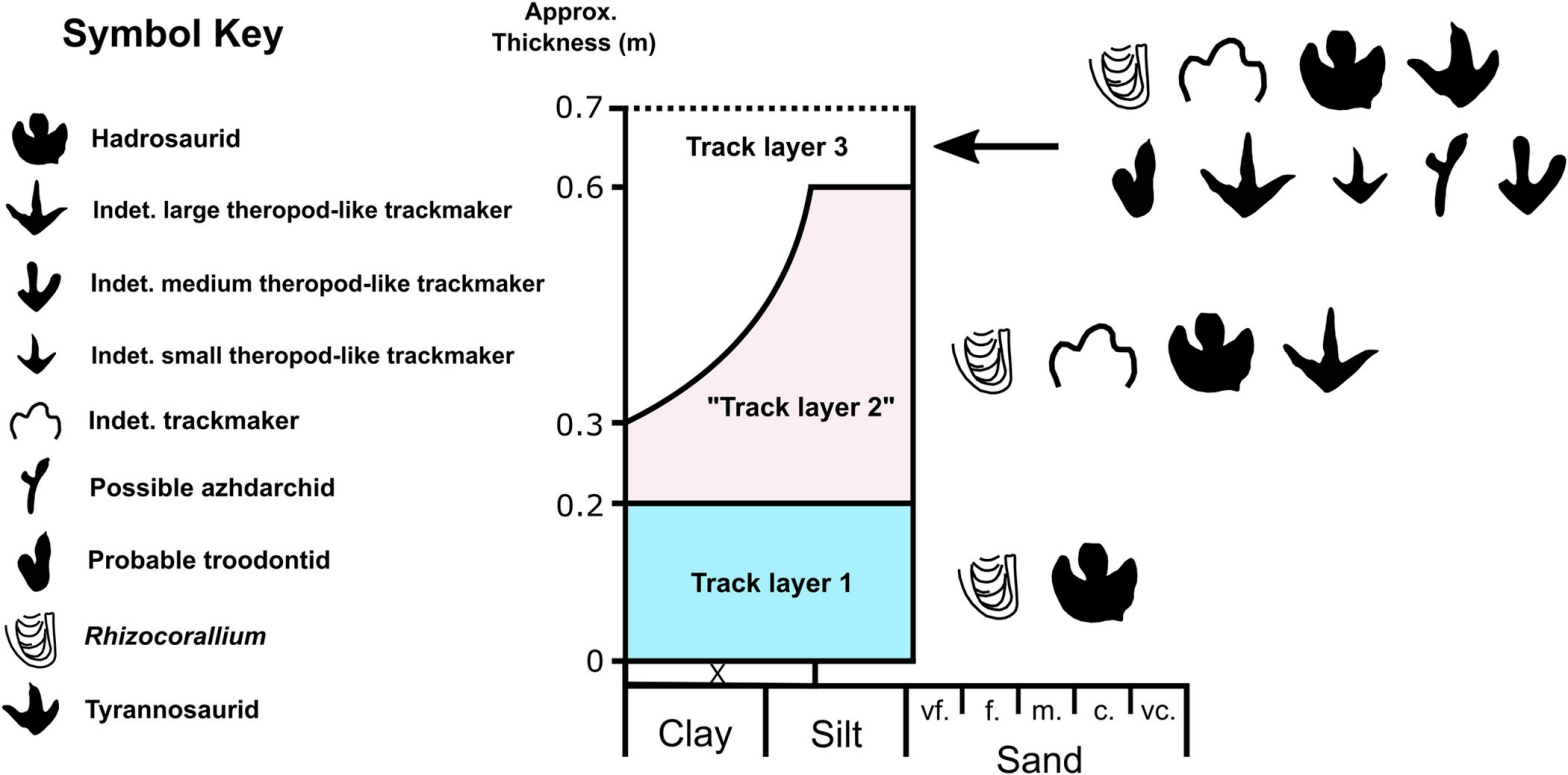
Localised stratigraphic column showing ichnofaunal diversity of each major track layer at Tyrants Aisle. Quotation marks around “track layer 2” reflect the likely presence of more than one distinct layer within this stratigraphic interval. Track layer colour scheme follows that of Figs 1B and 2–4.

At least three successive *in-situ* track-bearing horizons are present at Tyrants Aisle (Fig 6). The main track-bearing layers are numbered in stratigraphic order, 1 being the lowest and 3 the highest. Each has a strike that is approximately parallel with the flow of the modern Redwillow River (i.e., 235°), with an average sub-horizontal dip of 7° east.

**Fig 7:**
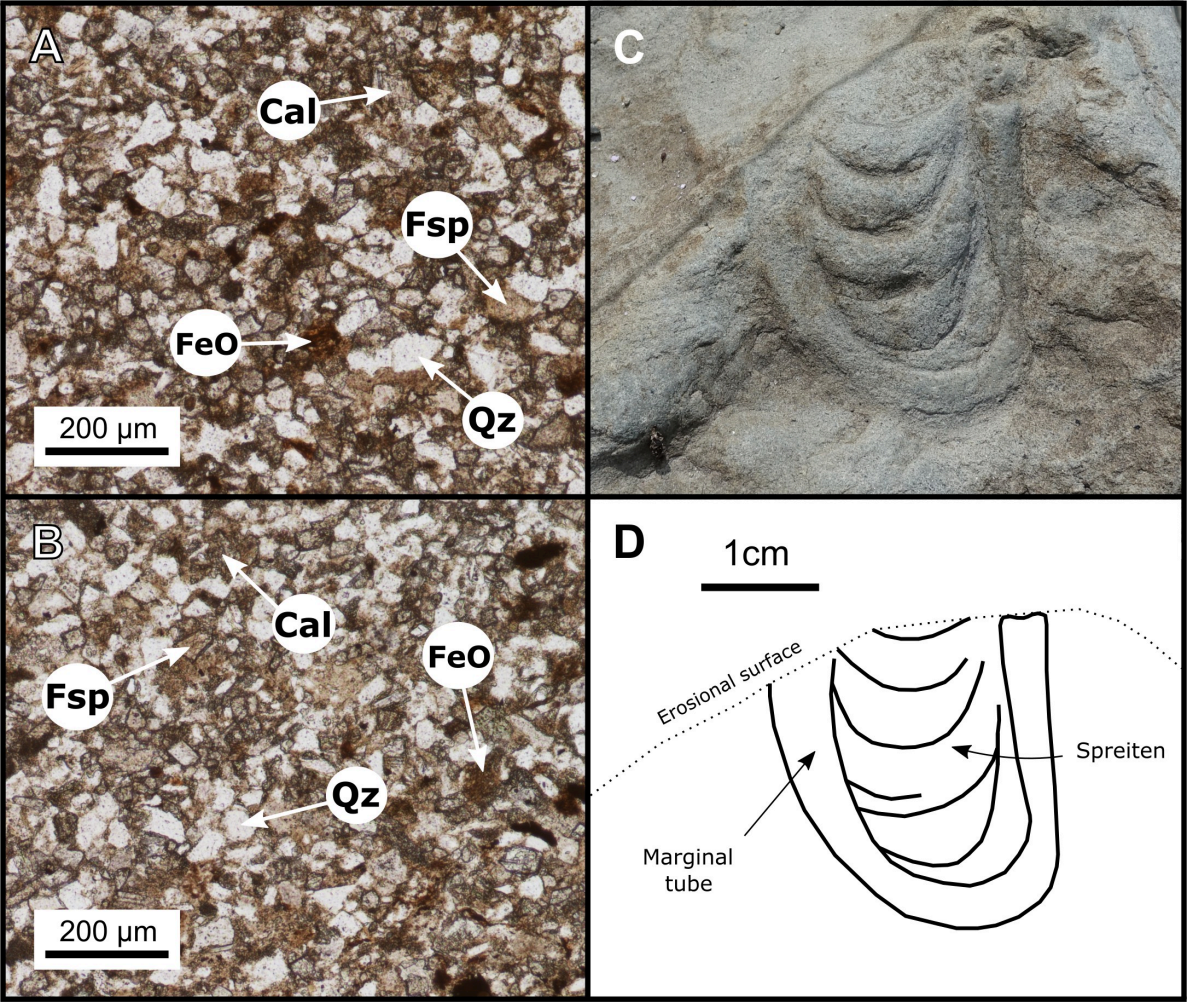
Petrographic thin sections of **A**, track layer 1 and **B**, track layer 3. **C**–**D**, Photograph and interpretive illustration of the most distinct *Rhizocorallium* invertebrate trace found at Tyrants Aisle, located within mapping grid 19-A on track layer 3 (Fig 1B). FeO = secondary iron oxide, while abbreviations for rock-forming minerals follow Whitney and Evans [71]: Cal = calcite, Fsp = feldspar, and Qz = quartz.

The three layers are petrographically similar in thin section: grains are well sorted and typically (>90%) below 120 μm in diameter (very fine sand to silt), but infrequently range up to ~250 μm (fine sand) (Figs 6, 7A and 7B). Clast mineralogy is dominated by quartz and feldspars (~50%, combined), which are embedded within a coarse calcitic cement (~40% of composition). Secondary iron oxides are also present (~5% of composition). Trace minerals (<1% of composition) include detrital zircons, tourmalines, chlorites, and muscovites. Clasts display low sphericity and range from angular to subrounded, the majority being subangular. These shape characteristics, in addition to the presence of elongate detrital muscovite fragments and crisp feldspars, suggest that the rocks are immature (i.e., grains had not travelled far from their source). Some grains of feldspar show partial replacement by calcite or chlorite, suggesting exposure to low heat, probably during shallow burial.

As fine-grained sediments are typically deposited under low energy, each track layer was likely laid down by slow-moving water. While Unit 4 of the Wapiti Formation is collectively a coal-rich unit [33, 40], no coal seams are present within the localised stratigraphic section at Tyrants Aisle, and the track layers contain relatively low organic content. Only one carbonised log fragment was observed on track layer 3 within grids 16E–17E (Fig 1B).

### Track layer 1

Layer 1 is exposed adjacent to the Redwillow River as a narrow band of outcrop ~0.2 m in vertical thickness and ranging from <1 m to 4.5 m in width across the length of the site. Tracks on this layer are restricted to metres 25–54 along the baseline (Figs 1B and 3). Only hadrosaurid tracks occur on this layer, and they are nearly all oriented at a bearing of ~120–150° and preserved as natural moulds.

### “Track layer 2”

Layer 2 exhibits variable geometry, extensive rock fracturing, and vertical displacement, resulting in a series of discontinuous ledges. The precise margins and lateral equivalences of these ledges are often difficult to determine. For simplicity, and due to the rarity of tracks on these surfaces, each ledge has been lumped together as a single area of outcrop situated between layers 1 and 3. It is probable that there is more than one distinct stratigraphic level contained within this interval. Downcutting of layer 2 caused its vertical thickness to become variable across the site, as the layer is shallowest towards the start of the mapping baseline (~0.1 m at grid number 8) and thickest near the far end of the site (~0.4 m at grid number 89). Two distinct track preservation styles were observed on layer 2 horizons: natural moulds were predominant, but there was at least one convex infilling (H.I.82C).

### Track layer 3

Layer 3 is the most extensively exposed stratigraphic layer at Tyrants Aisle. It is present across almost the entire length of the site and varies in exposed width from ~7 m to ~12.5 m. Vertical thickness of track layer 3 varies from ~0.1 to ~0.4 m across the site. Layer 3 is the main track-bearing horizon, with both the greatest quantity and diversity of dinosaur tracks, almost all of which are preserved as natural moulds (n≈169). Many of these natural moulds have been eroded into subcircular potholes, with few morphological details remaining. At least two *in-situ* tracks on layer 3, Th.Tw3.4.24H and H.I.74C, are preserved as convex infillings.

In addition to the tracks preserved on the three *in-situ* layers, at least three hadrosaurid tracks (H.I.3I; H.I.78C; H.I.81-D) are preserved as natural casts on *ex-situ* blocks that were evidently transported some distance by the Redwillow River. It is unclear which horizon these natural casts originate from.

Invertebrate ichnites are limited to traces of *Rhizocorallium*, identified based on their U-shaped burrow morphology and oblique orientation with respect to the bedding planes (Fig 7C and 7D) [72]. These ichnites were possibly produced by crustaceans, annelid worms or mayfly larvae [72], and occur sporadically as grouped clusters of ~2–6 on each of the three track layers (Figs 2 and 3). Not enough are exposed to determine if they are especially abundant within any particular layer (~15 were identified across all track layers). The burrows generally consist of a U-shaped marginal tube with interior spreiten (i.e., sedimentary laminae produced by the tracemaker while foraging and/or excavating), which join the two parallel ‘limbs’ of the marginal tube (Fig 7C and 7D), although some of the burrows lack visible spreiten. These invertebrate traces are most often observed in shallow marine facies, only rarely occurring in terrestrial strata [72]. No faecal pellets are visible within the burrow casts, which have been in-filled by the same sediments that form the rest of the track layers. *Rhizocorallium* are useful indicators of substrate consistency, as burrows containing actively-filled spreiten that differ in composition or texture to the marginal tube indicate relatively soft substrates. Conversely, burrows containing passively-filled spreiten that are identical to the in-filling of the marginal tube indicate relatively firm substrates [72]. As spreiten compositions within the *Rhizocorallium* at Tyrants Aisle are generally homogenous with respect to that of their marginal tubes (Fig 7C), passive in-filling is suggested, and the original substrates were likely relatively firm at the time of trace formation. However, as some dinosaur tracks on track layer 3 show evidence for suction effects and are relatively deep (see ‘indeterminate morphotype B’), a range of sediment consistencies from soft through to firm were apparently present at Tyrants Aisle, possibly reflecting variation in the timing of track formation.

Current directions can also be inferred from *Rhizocorallium*, as the parallel ‘limbs’ of the marginal tube are generally aligned (but oblique in the third dimension) with the prevailing current orientation [72–74]. *Rhizocorallium* burrows on track layer 1 are mostly aligned southeast–northwest, while those on track layer 3 are usually aligned north–south (Figs 2 and 3), consistent with prior observations that indicate a predominantly northward current flow within Unit 4 of the Wapiti Formation [33, 40]. Other palaeoflow indicators from Tyrants Aisle include ripple marks on track layer 3, although these are restricted to only a small area at the western end of the site (Fig 1B). The orientation of these ripples indicates a north-easterly direction of flow in this particular area. Given that the original rivers depositing sediments on the ancient Wapiti floodplains likely meandered to some degree, variability in flow direction is expected. Overall, an average northward flow is supported by both *Rhizocorallium* and ripple marks.

## Vertebrate ichnological diversity

Measurements of relatively well-preserved tracks and trackways at Tyrants Aisle are collated in Tables 1 and 2. To avoid repeating similar descriptions for multiple tracks, only the best-preserved examples with the most distinct and representative morphology are individually described for each trackmaker morphotype.

**Table 1:** Morphometric and spatial data for the best preserved or most representative individual tracks of each trackmaker morphotype.

Track number	Strat. layer	Track type	Pres.	CB3	Foot (L/R)	TL	TW	LD	RD	LL	LR	FLL	FL3	FLR	P3	HL	HR	BWL	BW3	BWR	MWL	MW3	MWR
H.Tw1.1.-2B	3	Hadrosaurid	Mould	270°	R	59.0	64.0	32°	52°	-	-	-	-	-	16.0	-	-	-	-	-	-	-	-
H.I.3I	*Ex-situ*	Hadrosaurid morphotype A	Cast	-	L?	64.0	64.0	24°	45°	57.5	56.0	11.0	20.0	12.0	15.0	48.5	48.5	18.0	32.0	18.0	10.0	20.0	12.0
H.I.9C	3	Hadrosaurid	Mould	104°	R?	50.0*	54.5*	30°	32°	44.0*	45.5*	7.5*	14.0*	10.0*	11.5*	37.5*	37.5*	14.5*	22.0*	19.5*	10.0*	16.0*	15.0*
H.I.19C	3	Hadrosaurid	Mould	152°	?	-	70.0	25°	29°	-	-	17.0	24.0	22.0	18.5	-	-	25.0	25.0	24.0	13.0	15.0	15.0
H.I.28-B	1	Hadrosaurid	Mould	256°	L?	56.0*	61.0*	10°	17°	51.0*	51.5*	24.0*	25.0*	22.0*	10.0*	29.5*	31.5*	21.0*	14.0*	22.0*	16.0*	20.0*	14.0*
H.I.28-C	1	Hadrosaurid	Mould	116°	R?	49.0*	53.0*	34°	41°	37.0*	38.5*	16.5*	27.0*	16.5*	17.5*	20.5*	22.0*	20.5*	8.0*	20.0*	16.0*	15.0*	11.5*
H.Tw3.1.30-C	1	Hadrosaurid	Mould	130°	R	50.5*	52.5*	43°	36°	41.0*	43.0*	11.0*	22.0*	15.0*	20.0*	33.0*	32.0*	21.5*	18.5*	24.0*	13.0*	15.5*	12.5*
H.Tw4.2.31-B	1	Hadrosaurid	Mould	149°	L?	49.5*	53.0*	38°	-	46.0*	-	17.5*	26.5*	-	-	31.0*	26.5*	17.5*	22.5*	-	11.5*	15.5*	-
H.Tw6.2.53A	3	Hadrosaurid	Mould	292°	R	62.0*	59.0*	26°	29°	54.0*	48.0*	14.0*	24.5*	10.0*	17.0*	38.5*	38.5*	22.5*	20.5*	14.0*	15.0*	14.5*	8.0*
H.Tw6.1.53B	3	Hadrosaurid	Mould	330°	L	59.0*	58.5*	25°	30°	53.0*	49.0*	16.0*	22.0*	12.5*	13.5*	37.0*	38.0*	20.0*	18.0*	19.0*	15.5*	16.5*	14.5*
H.Tw7.1.54A	3	Hadrosaurid	Mould	324°	R	59.5*	60.0*	31°	26°	56.5*	46.0*	16.0*	21.0*	7.0*	15.5*	40.0*	40.5*	19.0*	20.0*	20.0*	13.0*	17.5*	12.0*
H.I.68-A	3	Hadrosaurid	Mould	321°	?	62.0	66.0	34°	37°	58.5	49.0	17.0	25.0	13.5	17.0	44.0	35.0	22.0	21.0	20.5	15.0	19.0	13.0
H.I.72-A	3	Hadrosaurid	Mould	359°	L	53.0*	59.0*	36°	40°	44.0*	42.5*	7.5*	11.5*	6.0*	12.0*	39.0*	39.0*	16.5*	27.0*	15.0*	12.0*	16.5*	9.5*
H.I.72A	3	Hadrosaurid	Mould	314°	?	33.0*	-	-	35°	-	33.0*	-	8.0*	8.5*	-	28.0*	24.0*	-	15.5*	14.0*	-	9.5*	9.5*
H.I.76-B	3	Hadrosaurid	Mould	330°	?	56.0	57.0	49°	41°	49.0	37.5	18.0	25.0	15.0	23.0	41.0	30.5	16.0	27.0	18.0	7.0	16.0	11.0
H.I.78C	*Ex-situ*	Hadrosaurid morphotype A	Cast	-	?	65.0	70.0	67°	34°	54.0	54.0	18.0	20.0	13.5	19.0	-	48.0	-	26.0	23.0	-	16.5	14.0
Th.Tw1.4.6B	3	Ornithomimid or juvenile tyrannosaurid	Mould	059°	L	40.5	44.5	44°	48°	30.5	31.5*	19.0*	26.5	18.0	19.0*	14.0*	15.5*	9.0*	9.5*	8.0*	5.5*	5.5*	5.0*
Ty.I.8C	3	Tyrannosaurid	Mould	120°	R	49.0*	51.0*	44°	35°	40.0*	38.5*	19.5*	26.5*	15.5*	19.0*	23.5*	23.0*	13.0*	15.0*	10.0*	7.5*	11.0*	7.5*
Th.I.98-B	2	Ornithomimid or juvenile tyrannosaurid	Mould	274°	R?	40.5*	40.0*	-	-	-	-	14.0?*	28.5*	19.0?*	20.0?*	-	-	-	9.5*	-	-	5.5*	-
Ty.I.OG	*Ex-situ*?	Tyrannosaurid	Cast	011°	?	62.0	> 51	48°	-	45.5*	-	22.0	39.0	-	-	28.0	22.5	16.0	26.0	-	9.5	18.0	-
Di.Tw1.1.27D	3	Probable troodontid	Mould	252°	L	12.0	7.0*	32°	-	8.0*	-	3.0*	6.5*	-	-	5.0*	-	3.0*	2.0*	-	3.0*	3.0*	-
Di.I.34E	3	Probable troodontid	Mould	254°	L	13.0	7.5*	37°	-	8.0*	-	5.0*	10.0*	-	-	3.0*	-	1.5*	3.0*	-	1.5*	4.5*	-
Th.I.8C	3	Indet. medium theropod-like	Mould	165°	L	31.5*	30.5*	41°	65°	19.0*	19.0*	10.0*	23.0*	12.5*	19.0*	9.0*	9.5*	5.5*	7.5*	6.0*	3.5*	3.0*	4.0*
Th.Tw2.2.9C	3	Indet. small theropod-like	Mould	130°	R?	18.0*	14.0*	26°	31°	13.5*	16.0*	6.5*	12.5*	7.5*	6.5*	7.0*	8.5*	2.5*	7.5*	2.0*	1.5*	2.5*	1.5*
Th.I.10C	3	Indet. small theropod-like	Mould	154°	R?	12.5*	11.5*	40°	47°	6.5*	8.5*	3.5*	7.5*	4.5*	6.5*	5.0*	4.5*	1.5*	3.0*	2.0*	1.0*	2.0*	1.5*
Th.Tw5.3.72-A	3	Indet. small theropod-like	Mould	331°	R	18.0	17.0	36°	43°	13.5*	12.5*	6.5*	10.5*	5.5*	8.0*	7.5*	7.5*	4.0*	4.5*	4.0*	2.5*	1.5*	2.0*
Th.I.89-A	3	Indet. medium theropod-like	Mould	233°	?	35.0*	-	-	38°	-	31.0*	-	-	-	-	-	-	-	5.5	-	-	3.5*	-
Tri.I.4B	3	Indet. morphotype B	Mould	160°	?	32.0*	37.5*	57°	44°	24.5*	24.5*	-	16.0*	9.0*	17.0*	21.5*	16.5*	-	16.5*	12.0*	5.0*	6.5*	7.0*
Tri.I.5B	3	Indet. tridactyl	Mould	311°	?	26.0	25.5	30°	34°	22.5	18.0	9.5	12.5	6.0	9.0	13.0	14.0	5.0	6.0	5.5	5.0	4.5	4.0
Tri.I.8B	3	Indet. tridactyl	Mould	150°	L	36.0*	34.0*	31°	39°	29.0*	26.0*	12.5*	19.5*	10.0*	13.0*	17.5*	16.5*	9.5*	8.5*	8.0*	7.0*	9.0*	5.5*
Tri.I.10C	3	Indet. morphotype B	Mould	149°	?	17.0*	21.5*	48°	58°	13.5*	13.0*	-	7.5*	6.5*	8.5*	10.0*	10.0*	-	7.0*	-	2.5*	4.0*	2.5*
In.Tw1.1.76-A	3	Indet. morphotype A	Mould	-	L? (manus?)	28.5*	8.5*	-	-	-	-	-	-	-	-	-	-	-	-	-	-	-	-

Measurements adapted from Therrien et al. [43] and Salisbury et al. [44]. Strat. = stratigraphic context; Pres. = preservation type; CB3 = 360° compass bearing of the digit III midline (east from north); LD = divarication between the leftmost digit and digit III (in plan view); RD = divarication between the rightmost digit and digit III (in plan view). See Fig 5 for explanation of linear measurement abbreviations. Linear measurements are in cm and rounded to the nearest 5 mm. Asterisks (*) denote measurements taken digitally in Inkscape v. 0.92 from either a scaled photograph or digital elevation model.

**Table 2:** Trackway parameters for identified hadrosaurid and probable theropod sequences.

Trackway number	Trackway type	N	L/R	CBT	PL	SL	PA	FR	Hip height	Speed (m/s)
H.Tw1.-2B–2A	Hadrosaurid	3 (one missing)	L: (4?)	270°?*	1.29*: (3–4)	2.44?:	-	-	2.36 based on (1)	1.27?
			R: (1, 3?)			(1–3)				
H.Tw2.14B–12E	Hadrosaurid	3	L: (1, 3)	122°*	2.01*:	3.72*:	173.5°*	-3.5°*: (2)	2.40 based on (2)	2.52
			R: (2)		(1–2)	(1–3)				
					1.77*:					
					(2–3)					
H.Tw3.30-C–29-B	Hadrosaurid	2	L: (2)	130°*	1.22*	-	-	0°*: (1)	2.02 based on (1)	-
			R: (1)					-9°*: (2)		
H.Tw4.31-C–31-B	Hadrosaurid	2	L: (2)	130°*	1.13*	-	-	-20°*: (1)	1.98 based on (2)	-
			R: (1)					-19*: (2)		
H.Tw5.38B–31H	Hadrosaurid	1–5?	-	097°?*	-	-	-	-9°?*: (2)	2.70 based on (2)	-
H.Tw6.53B–53A	Hadrosaurid	2	L: (1)	297°*	1.10*	-	-	-33°*: (1)	2.48 based on (2)	-
			R: (2)					-5°*: (2)		
H.Tw7.54A–54-A	Hadrosaurid	2	L: (2?)	319°?*	0.96*	-	-	+5°?*: (1)	2.38 based on (1)	-
			R: (1?)					+3°?*: (2)		
H?.Tw9.69J–71G	Hadrosaurid?	1–4?	-	297°?*	1.17*:	2.64*:	175°*:	-	-	-
					(1–2)	(1–3)	(1–3)			
					1.46*:	2.54*:	173.5°*:			
					(2–3)	(2–4)	(2–4)			
					1.08*:					
					(3–4)					
H?.Tw10.71H–74J?	Hadrosaurid?	1–4?	-	-	1.23*, 1.46*, 1.11* (order unknown)	2.66*, 2.55* (order unknown)	-	-	-	-
Th.Tw1.9B–6B	Ornithomimid or juvenile tyrannosaurid	3 (one missing)	L: (2, 4)	056°*	1.27*:	2.58*:	-	-4°*: (1)	1.62 based on (4)	2.17
			R: (1)		(1–2)	(2–4)		-13°*: (2)		
								-2°*: (4)		
Th.Tw2.9B–9C	Indet. small theropod-like	2	L: (1)	109°*	0.89*	-	-	-16°*: (1)	0.72 based on (2)	-
			R: (2)					+19°*: (2)		
Th.Tw3.27D–24H	Indet. medium theropod-like	3–4?	L: (2, 4)	~110°*	1.67*:	3.55*:	-	+15°*: (1)	1.16 based on (1, 4)	5.46
			R: (1)		(1–2)	(2–4)		-1°*: (2)		
								+10°*: (4)		
Th.Tw4.71A–70-A	Indet. small theropod-like	4	L: (1, 3)	330°	0.47*:	0.76:	132°*:	+4°*: (2)	-	-
			R: (2, 4)		(1–2)	(1–3)	(1–3)	-9°*: (3)		
					0.36:	0.84:	149°*:	-6°*: (4)		
					(2–3)	(2–4)	(2–4)			
					0.49:					
					(3–4)					
Th.Tw5.71-A–72-A	Indet. small theropod-like	3	L: (2)	326°	0.49:	0.86	113°*	+18°*: (1)	0.72 based on (3)	0.89
			R: (1, 3)		(1–2)			-17°*: (2)		
					0.54:			+4°*: (3)		
					(2–3)					
Di.Tw1.27D–29D	Probable troodontid	1–4? (one or more missing?)	L: (1, 3?)	~257°*	-	-	-	-	0.48 based on (1)	-
			R: (2?)							

Abbreviations: N = number of prints preserved within trackway, with any missing tracks indicated in brackets; L = left foot; R = right foot; CBT = 360° compass bearing of the trackway midline (east from north); PL = pace length; SL = stride length; PA = pace angulation; FR = foot rotation, where (-) indicates inward rotation, and (+) indicates outward rotation. Data for L, R, PL, SL, PA, FR, and hip height are accompanied by bracketed numbers, which identify specific tracks within a particular trackway e.g., (3) identifies the third track within the trackway. Linear measurements are in metres. Asterisks (*) denote measurements taken digitally in Inkscape v. 0.92 from either a scaled photograph, map, or a still taken from a digital elevation model. Question marks indicate uncertain measurements, due to ambiguous print association, or cases where the foot which produced one or more tracks (i.e., left or right) is not certain. See Fig 5C and 5D for a visual demonstration of trackway measurements.

### *Hadrosauropodus* isp.

**Fig 8:**
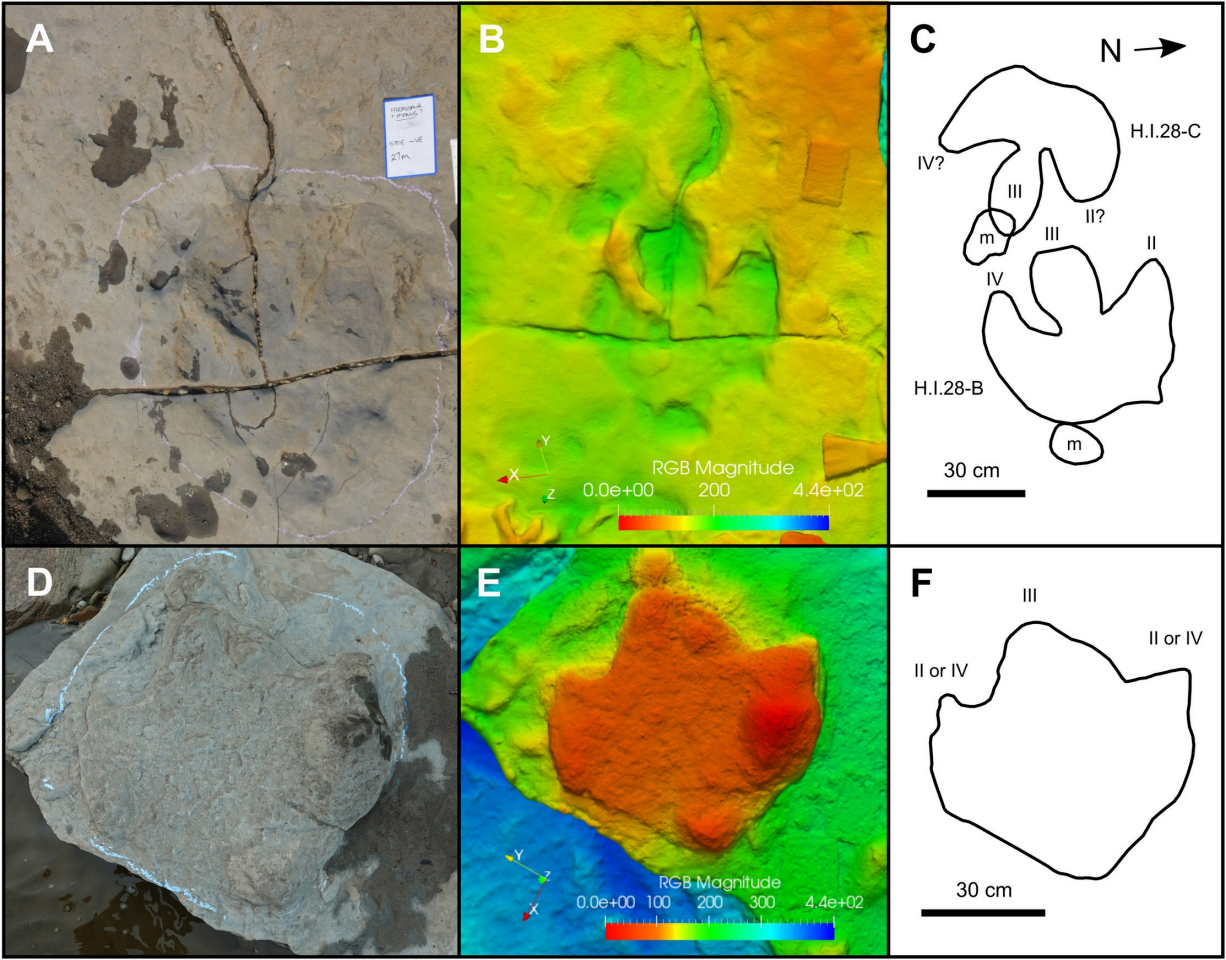
Representative hadrosaurid tracks from Tyrants Aisle. **A**, photograph, **B**, digital elevation model, and **C**, interpretive illustrations of *Hadrosauropodus* tracks H.I.28-B and H.I.28-C. The blue writing board measures 11.8 cm wide and 18.7 cm tall. Warm and cool colours indicate relatively high and low topography, respectively. **D**, photograph, **E**, digital elevation model, and **F**, interpretive illustration of hadrosaurid morphotype A track H.I.3I. Abbreviations: m = manus impression; II = digit II; III = digit III; IV = digit IV. All digital models—and the photographs used to create them—are available for download within the supplementary information.

H.I.28-B (exemplar print)—H.I.28-B is an isolated natural mould (Fig 8A–8C) (track length ≥56 cm; track width = 61 cm). The presence of an adjacent manus impression, which we assume is associated, anterolateral to digit III suggests that the track likely represents a left footprint [75]. The tip of digit III is not fully impressed. Digits II and IV gently taper to blunt points, with free lengths of 22 cm and 24 cm, and basal widths of 22 cm and 21 cm, respectively. Digit III is rounded, widest at its midpoint, and moderately constricted at the base. This basal constriction is also demonstrated in an adjacent isolated track (H.I.28-C; Fig 8A–8C), and in the holotype of *Hadrosauropodus* [75]. The heel impression is broad, with a weakly bilobed posterior margin. Other exemplars (e.g., H.Tw3.1.30-C and H.Tw4.2.31-B) are more prominently bilobed, and taper posteriorly into two small, blunt, typically obtuse points. Furthermore, some examples have entirely rounded heel impressions (e.g., H.Tw6.2.53A and H.Tw7.1.54A). These differences in heel shape are natural variants within *Hadrosauropodus* [75] and likely the result of variable substrate properties, kinematics, and preservation. In H.I.28-B, the divarication between digits II and III is 17°, while that between III and IV is 10°. The associated manus impression is a subrounded shallow depression (length = 11 cm; width = 18.5 cm), and overlies the tip of digit III in the adjacent track H.I.28-C. An additional subrounded manus impression is located immediately behind the heel base of H.I.28-B (Fig 8A–8C). It is unclear if this second manus imprint was created by the individual responsible for producing H.I.28-B, although this seems likely given its close proximity.

Less well-preserved *Hadrosauropodus* tracks are sometimes part of short trackway sequences (see ‘possible trackmaker sociality’). The poor preservation of these trackways precludes a detailed description of the individual tracks, although they remain useful for inferring the locomotory characteristics of the *Hadrosauropodus* trackmakers. The clearest examples on track layers 1 and 3 span only a single pace or stride length, and show predominantly inward foot rotation with respect to the trackway midline (Figs 1B, 2 and 3; Table 2). As the pace lengths are typically short (average of 1.35 m [n = 7]; Table 2), it is assumed that these *Hadrosauropodus* trackmakers were walking. Longer possible hadrosaurid trackways were identified on track layer 3 (Figs 3 and 4; Table 2), e.g., H.Tw5.38B–31H, although tracks within this sequence are ambiguous, comprising eroded depressions of variable size that can only tentatively be attributed to the same individual.

Remarks—Tanke [8] originally observed roughly a dozen ornithopod tracks at Tyrants Aisle in 2003, and referred these to *Amblydactylus*, an ichnogenus of probable hadrosauroid affinity containing two ichnospecies, *Amblydactylus gethingi* and *Amblydactylus kortmeyeri* (= *Caririchnium kortmeyeri* sensu [76]). Both were originally erected based on tracks from the Gething Formation (Aptian) of northeast British Columbia [77, 78]. This referral is not followed herein, given that tracks of *Amblydactylus gethingi* are longer than they are broad, while the heel impressions of the type series of *Amblydactylus kortmeyeri* are not prominently bilobed, and are generally convex in outline posterior to digit III [78]. In contrast, hadrosaurid tracks at Tyrants Aisle are almost always broader than they are long, and often have bilobed heel impressions, with a heel margin that is concave posterior to digit III (Fig 8A–8C).

*Hadrosauropodus langstoni*, the type ichnospecies of *Hadrosauropodus*, was originally erected based on TMP 1987.076.0006, a natural pes cast from the Maastrichtian horizons of the St. Mary River Formation in southern Alberta [60, 75]. Pes tracks generically referable to *Hadrosauropodus* are diagnosed as tridactyl, equally wide or wider than they are long, with rounded or bilobed heel margins wider than the proximal part of digit III, and broad digits with blunt distal terminations [75, 76, 79]. Within trackways, *Hadrosauropodus* pes tracks are generally rotated inward and have short pace lengths (approx. double track length). Smaller manus impressions are also sometimes associated with pes tracks [60, 75]. Based on the occurrence of the aforementioned characters in the majority of the hadrosaurid tracks at Tyrants Aisle (including H.I.28-B), as well as their appropriate geological age, we refer these tracks to *Hadrosauropodus* [75, 76, 79].

### Hadrosaurid morphotype A

H.I.3I (exemplar print)—H.I.3I is a large hadrosaurid pes natural cast located on an *ex-situ* block (Fig 8D–8F) (track length = 64 cm; track width = 64 cm). The heel is V-shaped, tapering to a blunt apex positioned in line with the axis of digit III. The free-length of each digit is short relative to the track length (left digit = 11 cm, digit III = 20 cm, right digit = 12 cm), pushing the hypices to a more anterior position than in typical examples of *Hadrosauropodus* [60, 75], and also greatly increasing the relative heel area of the print. As preserved, digit III is wider at its base than digits II and IV and tapers to a blunt, rounded termination. Divarication between digit III and the left digit is 24°, and that between digit III and the right digit is 45°.

Remarks—This hadrosaurid track morphotype is less abundant at Tyrants Aisle (n ≥2) than *Hadrosauropodus*, and is characterised by a digit III that widens towards the base (rather than being basally constricted as in H.I.28-B), a heel that is V-shaped rather than bilobed, and a proportionally larger heel area, features that create an overall more robust track morphology (Fig 8D–8F). Despite these morphological differences, it is assumed based on the provenance of the tracks (see ‘taxonomic affinities’) that the same trackmaker species was responsible for producing both these prints and those referred to *Hadrosauropodus*. Interestingly, the two best examples of this morphotype (H.I.3I and H.I.78C) are preserved as *ex-situ* natural casts, which suggests that preservation style may correlate with the occurrence of this morphotype.

### Tyrannosauridae

**Fig 9:**
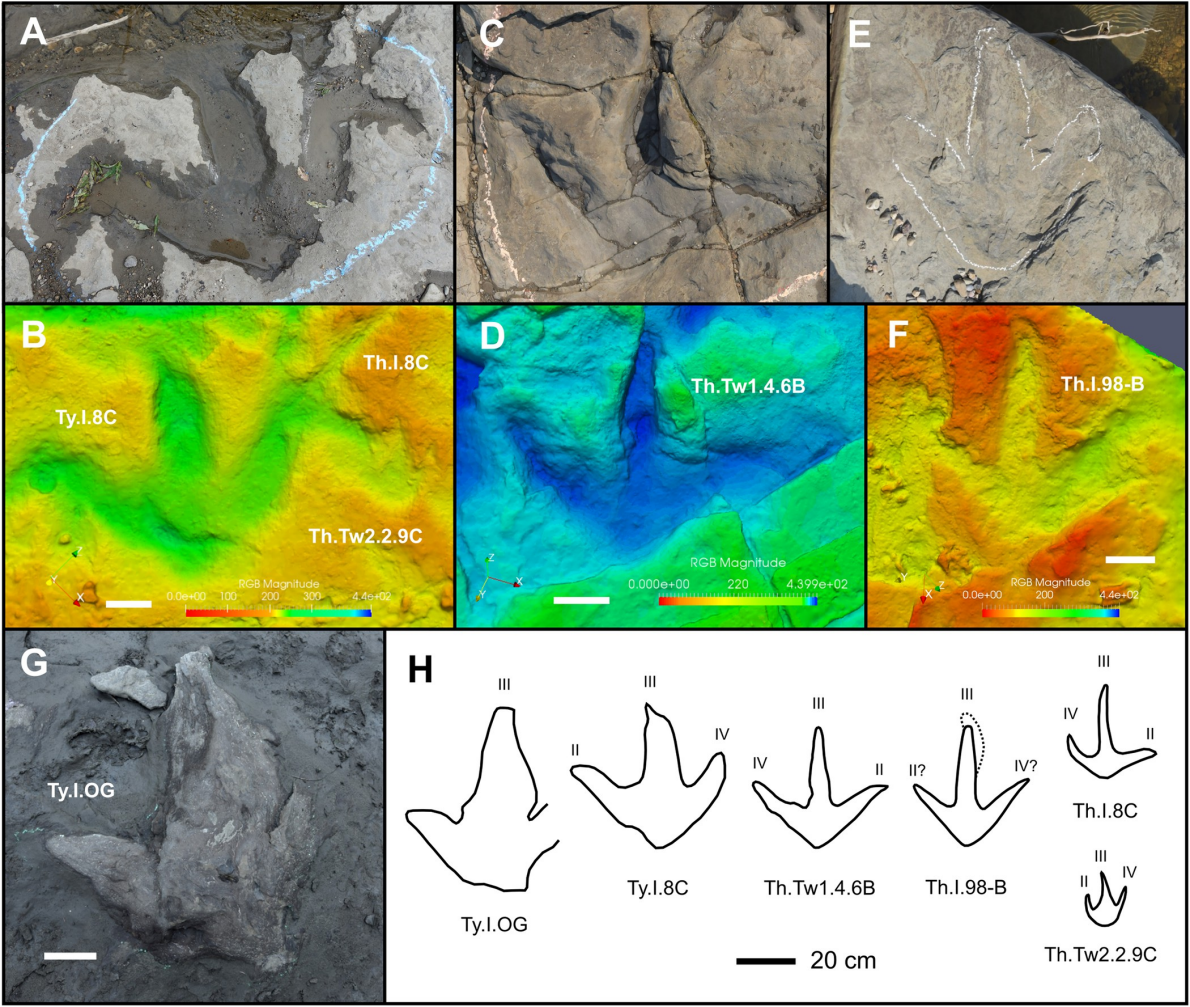
Tyrannosaurid and other probable theropod tracks. **A**, photograph and **B**, digital elevation model of tyrannosaurid track Ty.I.8C, medium theropod-like track Th.I.8C, and small theropod-like track Th.Tw2.2.9C. **C**, photograph and **D**, digital elevation model of cf. *Ornithomimipus* track Th.Tw1.4.6B. **E**, photograph and **F**, digital elevation model of large theropod-like track Th.I.98-B. **G**, photograph of large tyrannosaurid track Ty.I.OG, found ~40 m upstream of the mapped area. **H**, interpretive illustrations of depicted tracks. The dotted line indicates a possible drag mark or other continuation of the impression of digit III in Th.I.98-B. Scale bars = 10 cm unless otherwise indicated. Abbreviations: II = digit II; III = digit III; IV = digit IV. Images in A–D and outlines for Ty.I.8C and Th.Tw1.4.6B were modified from Fig 4 of Enriquez et al. [35]. All digital models—and the photographs used to create them—are available for download within the supplementary information.

Ty.I.OG (exemplar print)—The largest theropod track at Tyrants Aisle is an incomplete, isolated natural cast or convex infilling (track length≈62 cm; track width >51 cm) (Fig 9G–9H; Table 1) situated ~40 metres upstream from the mapped area. Although the specimen may be *in-situ*, loss of outcrop in the area between it and the main tracksite prevents Ty.I.OG from being assigned stratigraphically to any of the recognised track layers. It is difficult to determine which foot produced Ty.I.OG given that it is isolated, incomplete, and its morphology was substantially distorted by kinematics or poor preservation. Digit III is sinuous, unusually broad at the base (24 cm wide) and tapers to a blunt apex that is truncated by a break. The most dorsal surface of digit III forms a thin ridge that is most prominent towards the distal end. To the left of this ridge, the upper surface of digit III slopes downward at a shallow angle, whereas the right side slopes more steeply. The left digit diverges from digit III at an angle of 52°, and the free digit length of the former is less than half that of the latter (free length of left digit = 14.7 cm; free length of digit III = 38 cm). The right digit is truncated by a break, so that its length cannot be measured, although it appears to extend farther anteriorly than the left digit. Based on the remaining portion of the right digit, a divarication of ~35° can be estimated from digit III. Although incomplete, Ty.I.OG is strongly mesaxonic, with digit III projecting 34.5 cm beyond the left digit. The heel base is broad and tapers to a V-shaped apex, which is offset towards the right side of the track. Collectively, the offset heel and extreme length, width, sinuous form and asymmetrically sloped edges of digit III suggest that some sliding or other kinematic influence altered the shape of this track (see [64, 80] and references therein for further discussions of kinematic influence on dinosaur track morphology).

Remarks—Despite possibly being exaggerated in length, presumably due to trackmaker kinematics, Ty.I.OG is referable to a tyrannosaurid maker based on its large size (track length >45 cm; see ‘identification of trackmaker morphotypes’) and convex, high relief preservation that is inconsistent with an enlarged undertrack [62, 63]. As preserved, Ty.I.OG is equal in length to the holotype of the tyrannosaurid ichnospecies *Bellatoripes fredlundi* (i.e., print 2 of PRPRC 2011.11.001) [17]. The type material for *B*. *fredlundi* also occurs within lower Unit 4 of the Wapiti Formation, but was found further upstream along the Redwillow River, in the Tumbler Ridge area of British Columbia [17, 35]. *Bellatoripes fredlundi* tracks are diagnosed as being longer than wide, and having wide digit impressions that lack defined digital pads and are thick proximally but taper strongly distally [17]. In addition, the free length of digit III in *B*. *fredlundi* is relatively short, and the heel margin is relatively wide [17]. As the morphology of Ty.I.OG is distorted as a result of kinematic factors, this track cannot be convincingly referred to *B*. *fredlundi*. In particular, the relatively long free length of digit III in Ty.I.OG is inconsistent with the diagnosis of *B*. *fredlundi*.

An *in-situ*, isolated right theropod track preserved as a natural mould on track layer 3, Ty.I.8C, is also large enough to be confidently regarded as that of a tyrannosaurid (track length = 49 cm; track width = 51 cm) (Fig 9A, 9B and 9H). Ty.I.8C preserves low but well-defined track walls, several faint digital pad margins and sharp claw marks, indicating that it is not a deep undertrack with significantly exaggerated dimensions. Therefore, the length of this track can be taken at face value, and suggests it was produced by a tyrannosaurid ~1.95 m tall at the hip. Ty.I.8C lacks the features diagnostic of *Bellatoripes fredlundi*, including greater track length than track width, and digits that are wide proximally and taper strongly distally. Thus, Ty.I.8C is also not referable to *B*. *fredlundi*. For further description of Ty.I.8C see Enriquez et al. [35].

Regardless of ichnotaxonomic affinities, given their spatial and stratigraphic proximity, the same tyrannosaurid species possibly produced tracks Ty.I.OG, Ty.I.8C, and those of *B*. *fredlundi* at their type locality in British Columbia [17]. Morphological differences between these tracks may be partly explained by ontogeny [35].

### Indeterminate small theropod-like tracks

**Fig 10:**
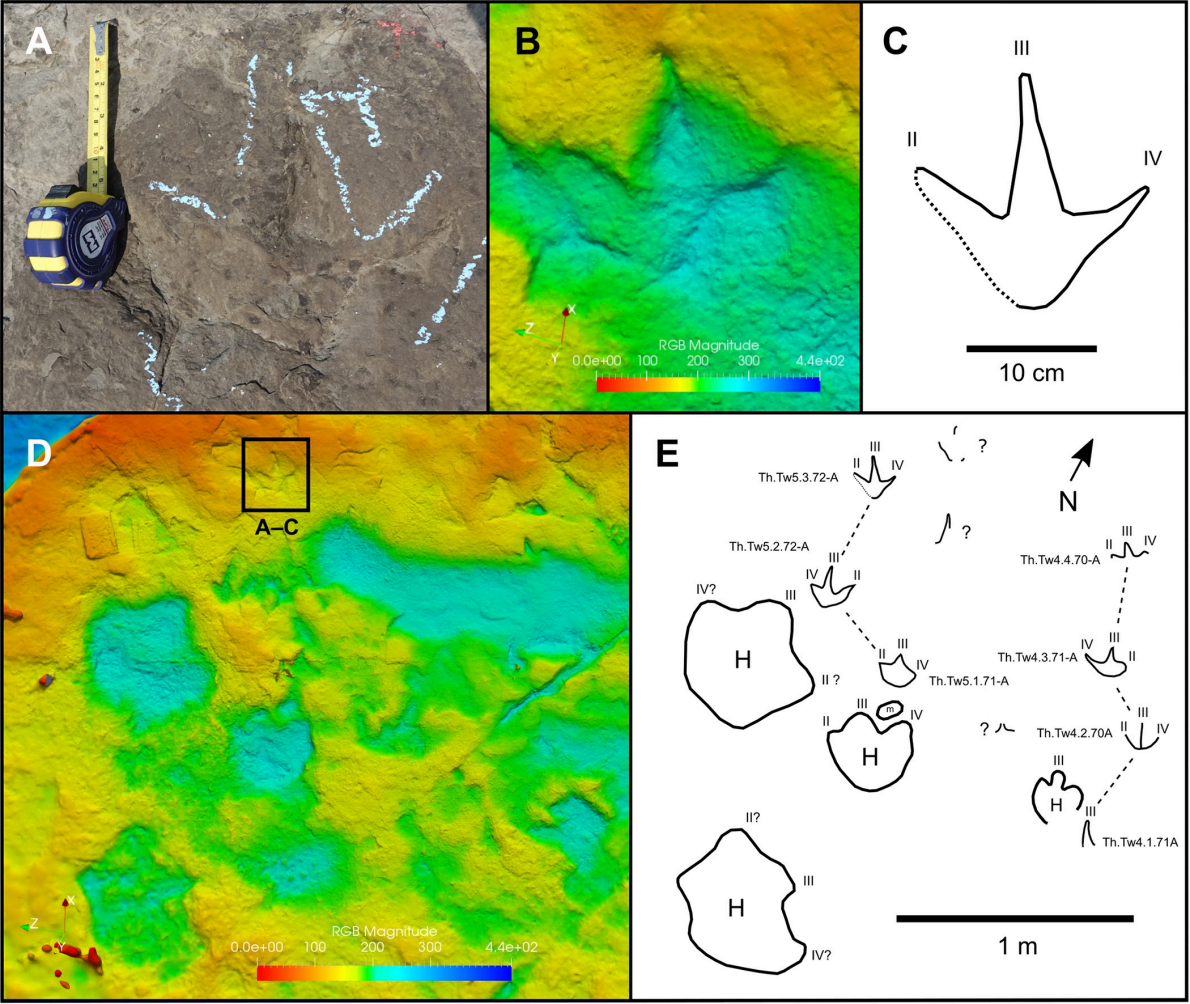
Parallel, small, tridactyl theropod-like trackways. **A**, photograph, **B**, digital elevation model, and **C**, outline drawing of track Th.Tw5.3.72-A. **D**, Digital elevation model, and **E**, outline drawing of the area of track layer 3 containing adjacent small theropod-like trackways Th.Tw4.71A–70-A and Th.Tw5.71-A–72-A, in addition to prints of hadrosaurids. The digital model in B and D—and the photographs used to create it—are available for download within the supplementary information.

Th.Tw5.3.72-A (exemplar print)—Th.Tw5.3.72-A is the best-preserved and last track within a trackway of three consecutive footprints (Th.Tw5.71-A–72-A; Fig 10; Table 2). A silicone mould of this trackway is accessioned as UALVP 59920. Th.Tw5.3.72-A is longer than wide (track length = 18 cm; track width = 17 cm; length/width ratio = 1.06), and is preserved as a natural mould on track layer 3 (Fig 10A–10C). Digit III is widest at the base (greatest width = 4 cm) and tapers steadily until the most distal third, at which point the digit tapers more gradually. This track was produced by a right foot, given its placement relative to preceding tracks in the sequence (Fig 10D and 10E). Divarication between digits II and III is 36°, while that between III and IV is 43°. The heel impression tapers to a blunt V-shape that forms a ~90° angle, although the preceding tracks within the same sequence show more gently rounded heels, which probably reflects variation in substrate properties, kinematics or preservation.

Trackway parameters for Th.Tw5.71-A–72-A—A step length of 0.49 m separates Th.Tw5.1.71-A from Th.Tw5.2.72-A, followed by a second step of 0.54 m between Th.Tw5.2.72-A and Th.Tw5.3.72-A (Table 2). A stride of 0.86 m occurs between Th.Tw5.1.71-A and Th.Tw5.3.72-A (Fig 10D and 10E; Table 2). Hip height of the trackmaker is estimated at 0.72 m, based on the length of Th.Tw5.3.72-A. From these measurements, the trackmaker is inferred to have been moving at a speed of 0.89 m/s (3.22 km/h). The stride length:hip height ratio is 1.19, indicating a walking gait [6]. Similar track size, pace and stride length observed in an adjacent and parallel small theropod-like trackway, Th.Tw4.71A–70-A, suggests that the two trackmakers may have been walking together at a similar speed (Fig 10D and 10E; Table 2) (see ‘possible trackmaker sociality’).

Remarks—At least 11 tridactyl theropod-like tracks occur within the smallest indeterminate size class, and these tracks are morphologically similar to one another. The smallest example, Th.I.10C, measures 12.5 cm in length and—along with the probable deinonychosaur tracks Di.Tw1.1.27D and Di.I.34E—is among the smallest non-avian dinosaur tracks documented from the Wapiti Formation (Figs 2 and 3; Table 1). At least two small tridactyl theropod-like tracks are isolated, while the rest occur within three (or possibly four) short trackway sequences (Figs 1B, 2 and 10).

### Indeterminate medium theropod-like tracks

**Fig 11:**
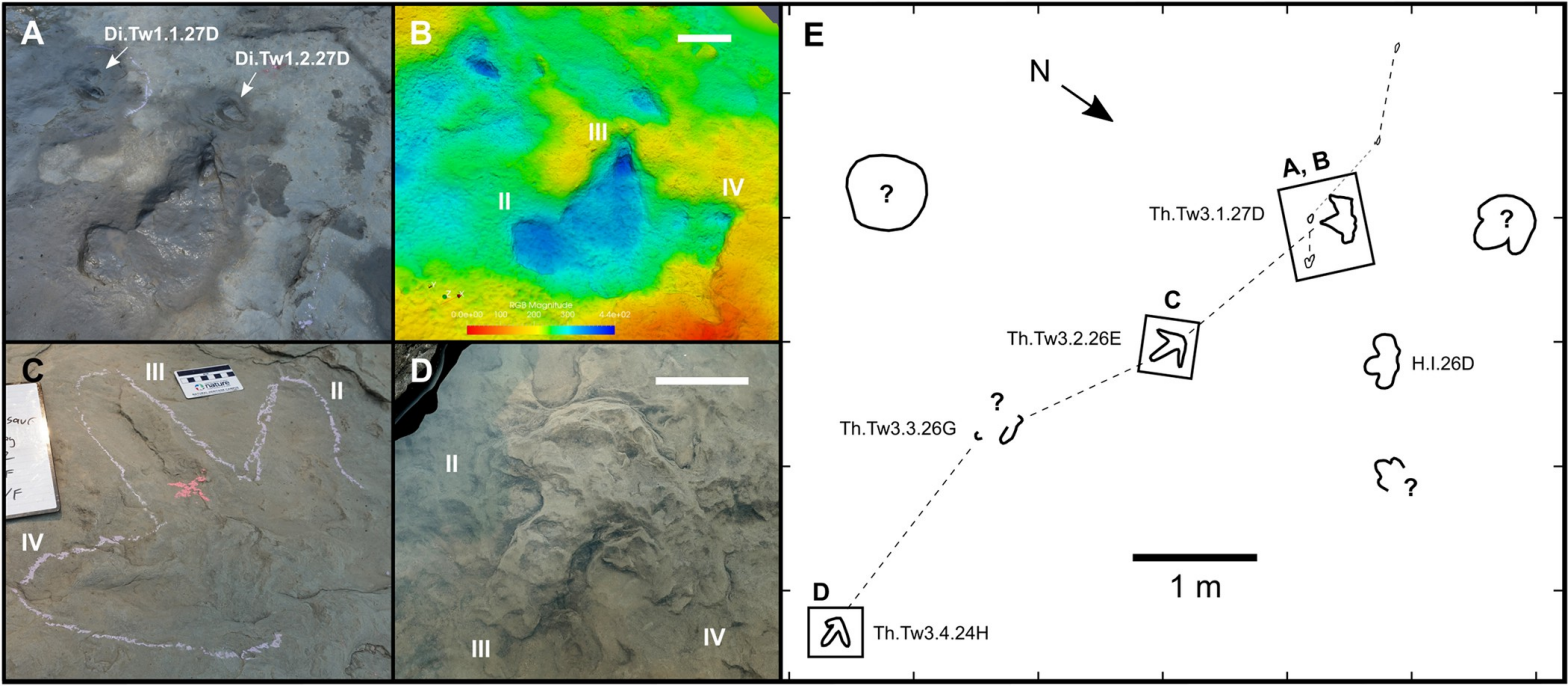
Medium-sized probable theropod trackway (Th.Tw3.27D–24H) and probable troodontid tracks (Di.Tw1.1.27D and Di.Tw1.2.27D) on track layer 3. **A**, photograph and **B**, digital elevation model of Th.Tw3.1.27D, Di.Tw1.1.27D and Di.Tw1.2.27D. **C**, photograph of Th.Tw3.2.26E. **D**, photograph of Th.Tw3.4.24H. **E**, interpretive map illustration of trackway area. Images and outlines in A–B and D–E were modified from Fig 3 of Enriquez et al. [37]. Scale bars = 10 cm, unless otherwise indicated, while the physical scale shown in C = 8.2 cm. The digital model in B—and the photographs used to create it—are available for download within the supplementary information.

Th.Tw3.27D–24H (exemplar trackway)—Th.Tw3.27D–24H is a sequence of three (or possibly four) tracks, which are highly variable in morphology and preservation (Fig 11). The first track in the sequence, Th.Tw3.1.27D (track length = 30 cm; track width = 40.5 cm), is relatively clear and possesses well-defined, short track walls (Fig 11A and 11B). Based on its position relative to subsequent footfalls, Th.Tw3.1.27D is a right print. The shape of Th.Tw3.1.27D is highly irregular: digit II is short and rounded, while digits III and IV are longer and more pointed. In addition, the heel impression is short, with a posterior margin that is almost straight and perpendicular to the digit III axis, making the track considerably wider than it is long (length:width ratio = 0.74). The second track, Th.Tw3.2.26E, occurs 1.7 m ESE from Th.Tw3.1.27D and is a poorly defined left print, comprising three faint, very shallow and highly eroded depressions that correspond to the expected positions of the digits (Fig 11C). However, the distal end of digit IV is clearly visible and terminates in a sharp, narrow claw mark. A possible third track, Th.Tw3.3.26G, is located 1.15 m ESE of Th.Tw3.2.26E, although it is difficult to confirm due to a high level of erosion. The last track in the sequence, Th.Tw3.4.24H, is preserved as a convex infilling (track length = 28 cm) (Fig 11D). Th.Tw3.4.24H is 3.55 m ESE of Th.Tw3.2.26E, and represents a left track based on its position within the trackway. The heel margin of Th.Tw3.4.24H forms a blunt V shape, suggesting that the relatively straight heel margin of Th.Tw3.1.27D is a preservational artefact. Digit II of Th.Tw3.4.24H is truncated, while digits III and IV, although waterworn, are nearly complete and possess rounded, blunt terminations. Based on the stride length of 3.55 m between the second and fourth tracks, and using the average track length of Th.Tw3.1.27D and Th.Tw3.4.24H ($\bar{x}$ = 29 cm) as a rough foot length approximation, the trackmaker was moving at ~5.46 m/s (~19.65 km/h). A stride length:hip height ratio of 3.06 suggests a running gait [6]. However, this interpretation is tentative as the track size and, by extension, the hip height may be an underestimate, given that the lengths of Th.Tw3.1.27D and Th.Tw3.4.24H both appear to have been reduced by poor preservation and erosion.

Remarks—In addition to Th.Tw3.27D–24H, five isolated tracks on track layer 3 fall into the medium indeterminate theropod-like size class: Th.I.8C, Th.I.62H, Th.I.65G, Th.I.74J, and Th.I.89-A. The long, slender digit proportions of some of these tracks, particularly Th.I.8C, are comparable to those seen in larger specimens such as Th.Tw1.4.6B and Th.I.98-B (Fig 9), which may pertain to distinct size classes of the same trackmaker.

### Indeterminate large theropod-like tracks

Th.I.98-B (exemplar print)—Located on track layer 2, Th.I.98-B is an isolated large theropod track (track length≈40.5 cm; track width = 40 cm; length:width ratio≈1.01) preserved as a shallow, eroded natural mould (Fig 9E, 9F and 9H). Digit impressions are long, slender, and gently tapering. A shallow, curving trace at the distal end of digit III may be a toe drag or, alternatively, is unrelated (Fig 9E, 9F and 9H). If the curving trace records the distal curvature of digit III, the track is likely a right footprint, as the tip of digit III in theropod tracks is usually in-turned medially [81–83]. Divarication between digits II–III and III–IV is 42°. The metatarsophalangeal area is small relative to the track length, although the posterior heel apex is ambiguous and difficult to identify due to erosion. No interphalangeal pads are visible.

Remarks—Tridactyl theropod-like footprints in this upper size class almost certainly pertain to theropod trackmakers rather than thescelosaurids. This conclusion is based on expected body size; the largest known thescelosaurid species—*Thescelosaurus garbanii* from the upper Maastrichtian Hell Creek Formation of Montana*—*possesses a pes approaching 30 cm in length (based on LACM 33542), while other members of the clade are considerably smaller [84]. The two most exemplary of these tracks, Th.Tw1.4.6B and Th.I.98-B, both possess relatively long, slender digit impressions and are almost identical in size (Fig 9). Th.Tw1.4.6B is the final footprint within the trackway Th.Tw1.9B–6B, and was previously described in detail by Enriquez et al. [35]. Th.Tw1.9B–6B was tentatively referred to cf. *Ornithomimipus* based on slender digit proportions, constriction near the base of digit III in Th.Tw1.4.6B, and visible separation of digit II from the rest of the track in Th.Tw1.2.8B [35, 85–87]. Although tracks assigned to *Ornithomimipus* are generally treated as the footprints of ornithomimosaurs [85–87], the trackmaker of Th.Tw1.9B–6B was possibly a juvenile tyrannosaurid [35]. Th.I.98-B demonstrates similarly slender digit proportions to Th.Tw1.4.6B, but we refrain from also referring this track to cf. *Ornithomimipus* in the absence of a well-defined constriction near the base of digit III or clear separation of the impression of digit II from the rest of the track.

### Deinonychosauria

Di.Tw1.1.27D and Di.I.34E (exemplar prints)—Di.Tw1.1.27D and Di.I.34E were previously described in detail and figured by Enriquez et al. [37]. Thus, only a brief reiteration of their basic morphology is presented here. Di.Tw1.1.27D and Di.I.34E measure 12 and 13 cm in length, and 7 and 8 cm in width, respectively (Table 1). Digit III is broad and elliptical in both tracks, whereas digit IV is relatively short (IV:III length ratio = 0.6–0.68) and rounded in Di.Tw1.1.27D (Fig 11A, 11B and 11E), but more arcuate in Di.I.34E. The heel margins of both tracks are rounded, and there is no evidence of digit II, or of any digital pad impressions except a proximal pad margin at the base of digit III in Di.I.34E. Based on track lengths, hip heights of 0.48 m and 0.52 m are estimated for the trackmakers of Di.Tw1.1.27D and Di.I.34E, respectively.

Remarks—Di.Tw1.1.27D and Di.I.34E constitute the first probable deinonychosaur tracks from Canada [37]. The relative shortness of digit IV indicates that both were likely produced by troodontid trackmakers, rather than dromaeosaurids [37, 88, 89]. Di.Tw1.1.27D and Di.I.34E are spaced ~6.5 m apart and, although both are oriented at a bearing of 252–254°, their lateral separation suggests they were produced by different individuals (Fig 3). Di.Tw1.1.27D may instead be associated with three additional, predominantly monodactyl impressions: Di.Tw1.2.27D, Di.Tw1.3.28D, and Di.Tw1.4.29D, collectively identified as Di.Tw1.27D–29D (Figs 3, 11A, 11B and 11E). However, suboptimal preservation, differences in digit counts, and abnormalities in relative spatial positioning make this collective trackway designation tentative [37]. The variation between didactyly and monodactyly within Di.Tw1.27D–29D is likely related to differential levels of track erosion, while missing footprints or the presence of additional trackmakers may explain their spatial abnormalities [37].

### Indeterminate morphotype A

**Fig 12:**
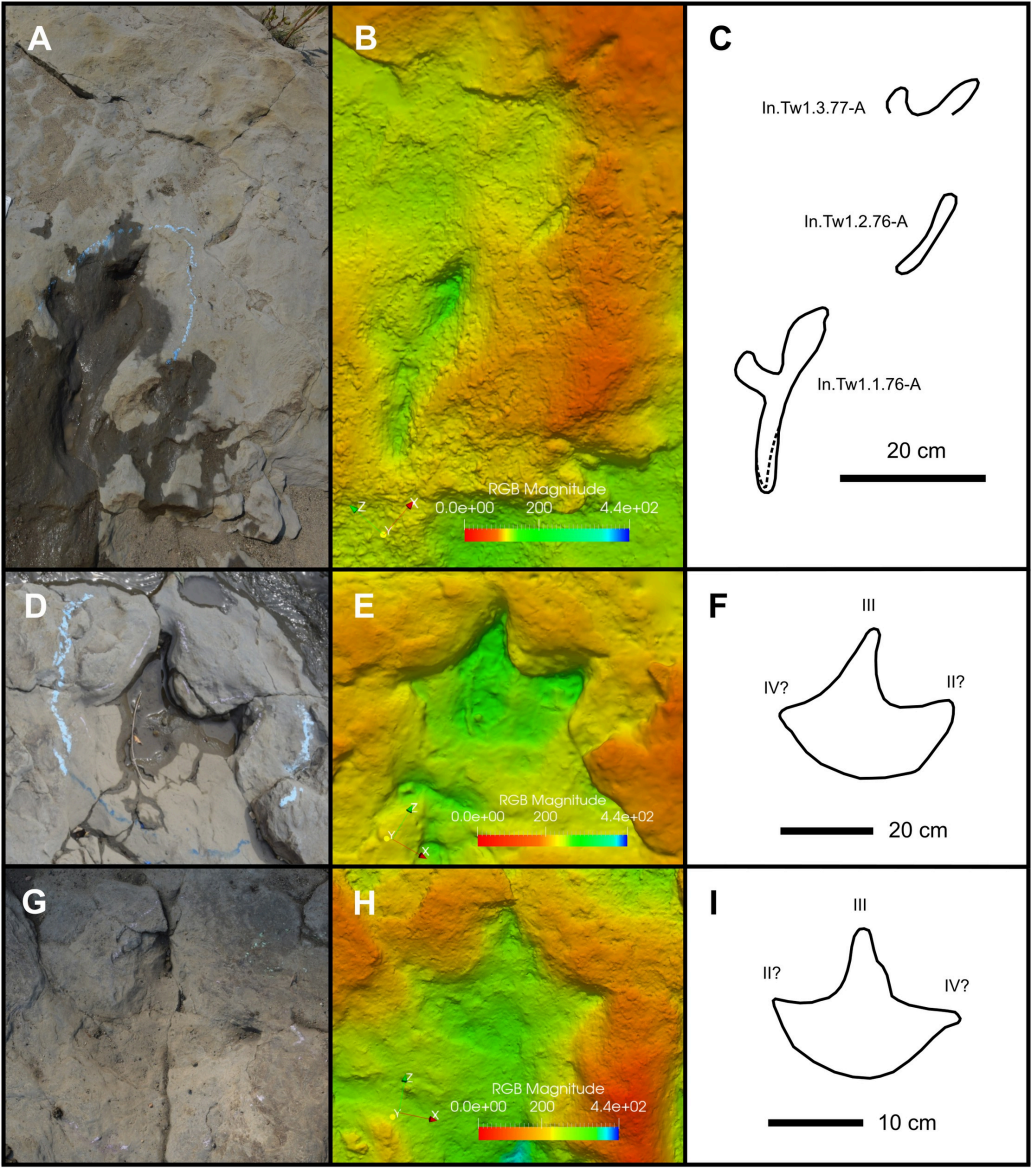
Traces of uncertain affinity. **A**, photograph, **B**, digital elevation model, and **C**, outline drawing of the indeterminate morphotype A trackway In.Tw1.76-A–77-A, tentatively assigned to an azhdarchid pterosaur. **D**, photograph, **E**, digital elevation model, and **F**, outline drawing of indeterminate morphotype B track Tri.I.4B. **G**, photograph, **H**, digital elevation model, and **I**, outline drawing of indeterminate morphotype B track Tri.I.10C. All digital models—and the photographs used to create them—are available for download within the supplementary information.

In.Tw1.76-A–77-A (exemplar trackway)—Three elongate traces follow one another in close succession on track layer 3 (Fig 12A–12C), the first of which (In.Tw1.1.76-A) is the largest and most distinct. In.Tw1.1.76-A measures 28.5 cm and 8.7 cm in greatest length and width, respectively. The longest portion of the trace consists of a narrow, trench-like impression that is both deepest and widest at the presumed anterior end, and comparatively shallow and narrower at the posterior end (Fig 12A–12C). A shorter lateral impression diverges at ~90° from near the midpoint of the main trace, the tip of which is curved anteriorly. Raised topography near the middle of the main trace (immediately adjacent to where the shorter impression diverges) probably represents minor mud collapse or partial infill. The second trace in the sequence, In.Tw1.2.76-A, is poorly preserved and appears only as a narrow, shallow furrow that is eroded and more distinctly visible in the digital elevation model (Fig 12B) than the corresponding photo (Fig 12A). There is no evidence of a shorter, laterally divergent impression like that present in In.Tw1.1.76-A. The third and final trace, In.Tw1.3.77-A, is also heavily eroded and best viewed from the digital elevation model (Fig 12B). As preserved, only part of the trace is recognisable, which is nearly identical to the anterior portion of In.Tw1.1.76-A. The anterior end of In.Tw1.3.77-A is the most deeply impressed part of the trace, and there is evidence of a shorter, laterally diverging impression that curves anteriorly and terminates in a rounded tip. The striking similarity between In.Tw1.1.76-A and In.Tw1.3.77-A, as well as their close association, indicates that the observed morphology is real and not a product of track surface erosion. No other traces with a similar morphology were observed on any of the track layers.

Remarks—Tracks assigned to indeterminate morphotype A are not morphologically consistent with those that any of the previously identified dinosaurian trackmakers would be expected to produce while engaging in ‘normal’ terrestrial locomotion. Swimming dinosaurs may produce tracks with atypical morphologies, including parallel sets of sinuous scratches, the presence of kick-off scours, and raised mounds of sediment at the posterior end of the track [90, 91]. As these features are lacking in indeterminate morphotype A tracks at Tyrants Aisle, and indeed are absent among tracks across the whole locality, the probability that tracks of this morphotype are swim traces is low. Alternatively, the long, narrow, posterior trace within In.Tw1.1.76-A could represent a metatarsal impression of a dinosaurian trackmaker that sank in soft sediment, or was crouching as it walked [92]. However, dinosaur tracks preserving metatarsal impressions generally also retain impressions of their weight-bearing pedal digits at the anterior end of the trace [92]. Only a single impression occurs at the anterior ends of In.Tw1.1.76-A and In.Tw1.3.77-A, which is inconsistent with a tridactyl dinosaurian trackmaker.

In.Tw1.1.76-A and In.Tw1.3.77-A resemble some pterosaur manus tracks [93–96], both in their elongate, narrow form and in the presence of a shorter impression that diverges from the longest furrow axis (digit II in most pterosaur manus tracks). If In.Tw1.1.76-A was produced by a pterosaur, its large size suggests an azhdarchid trackmaker, which is consistent with its late Campanian age. Skeletal remains of azhdarchids are rare in western Canada, and presently limited to the Oldman and Dinosaur Park formations (mid–late Campanian) of southern Alberta [97–102], and possibly also the Northumberland Formation (Campanian) of Hornby Island, British Columbia [103]. Nevertheless, another possible large pterosaur manus track (TMP 1987.55.39) was previously described from lower Unit 4 of the Wapiti Formation by Bell et al. [9]. TMP 1987.55.39 is preserved as a natural cast on an isolated boulder and was collected from Pinto Creek, ~30km from Tyrants Aisle [9]. TMP 1987.55.39 measures 25.5 cm in length, slightly smaller than In.Tw1.1.76-A. Both tracks consist of a relatively long, narrow trace that is nearly perpendicular to a shorter trace, which diverges from the former close to its midpoint. However, unlike in TMP 1987.55.39, the shorter traces of In.Tw1.1.76-A and In.Tw1.3.77-A are curved, presumably in the anterior direction. Furthermore, in TMP 1987.55.39, the longer trace (i.e., the aligned impressions of digits I and III) is curved towards the shorter, perpendicular trace (i.e., the impression of digit II). By contrast, the longer trace in In.Tw1.1.76-A curves away from the presumed digit II impression (Fig 12A–12C). These differences do not necessarily suggest distinct trackmakers, as in some other azhdarchid manus prints (e.g., the holotype manus track of *Haenamichnus uhangriensis*, CNUPH.P2 [93]), the axis of the longer trace has a similar direction of curvature to that seen in In.Tw1.1.76-A.

Thus, an azhdarchid pterosaur is tentatively suggested as the trackmaker of indeterminate morphotype A. This occurrence is notable as the trackway In.Tw1.76-A–77-A and the isolated, previously described track TMP 1987.55.39 presently constitute the only possible evidence of azhdarchids within the Wapiti Formation.

### Indeterminate morphotype B

Tri.I.4B (exemplar print)—Tri.I.4B occurs on track layer 3 and measures 32 cm in length and 37.5 cm in width (Fig 12D–12F; Table 1). Its three digit impressions are deeply impressed into the substrate, whereas the rounded heel margin is shallower and more extensively eroded. Digit III curves to the right in plan view, suggesting that Tri.I.4B may be a left footprint. Divarication between digits III and IV is 57°, and 44° between digits II and III (Table 1). Digits II and IV are relatively short, broad at the base, and the free portion of these digits are difficult to distinguish from the rest of the track.

Remarks—Three isolated specimens are assigned to this morphotype (Tri.I.4B, Tri.I.6A and Tri.I.10C), which were probably made by a hadrosaurid or theropod. Indeterminate morphotype B is characterised by tridactyl prints with semi-circular, rounded heel margins and relatively short, broad digits II and IV that taper strongly and are nearly ‘closed’ (i.e., lacking a defined free length) (Fig 12D–12I). Such a pattern of digit morphology suggests that the creation of this morphotype involved track wall collapse or suction during withdrawal of the foot from the substrate. Similar tracks have also been observed elsewhere (e.g., Fig 6A of Razzolini et al. [104]), and their occurrence at Tyrants Aisle indicates that at least some of the tracks at the site were formed when the substrate was soft and relatively water saturated.

## Discussion

### Taxonomic affinities

Large ornithopod tracks from Tyrants Aisle are attributed to hadrosaurids, whose skeletal remains and tracks are ubiquitous throughout the Wapiti Formation [8, 10–12, 15–17, 55, 105]. Within Unit 4, a ‘mummified’ specimen of the saurolophine *Edmontosaurus regalis* (UALVP 53722) was found near Red Willow Falls, ~16km upstream from Tyrants Aisle [15]. This specimen occurred two metres below a bentonite layer dated to 72.58±0.09 Ma [15]. As Tyrants Aisle is at roughly the same stratigraphic level within Unit 4 (or slightly stratigraphically higher, see ‘geographic and geological setting’), and is a relatively short distance away (Fig 1A and 1C), it seems likely that the hadrosaurid trackmakers at Tyrants Aisle were also individuals of *Edmontosaurus regalis*. This is further supported by the temporal correlation between Unit 4 of the Wapiti Formation and the lower members of the Horseshoe Canyon Formation in southern Alberta [34, 39, 42], in which *E*. *regalis* is the sole hadrosaurid [106].

Tridactyl theropod-like footprints at Tyrants Aisle greater than 45 cm in length can be attributed with confidence to tyrannosaurids (see ‘identification of trackmaker morphotypes’). The presence of tyrannosaurid tracks is significant, as their body fossil record within the Wapiti Formation is presently restricted to teeth, an isolated vertebra (TMP 1989.062.0004), and a metatarsal (TMP 2005.066.0047) [10, 12, 14, 54, 105]. Neither TMP 1989.062.0004, TMP 2005.066.0047, nor any of the isolated teeth are generically identifiable. However, the most spatiotemporally proximate species is *Albertosaurus sarcophagus*, recovered from the Danek Bonebed (~71.8–71.5 Ma) in the Horsethief Member of the Horseshoe Canyon Formation, near Edmonton, ~450 km SE of Tyrants Aisle [24–25, 34, 42, 107]. Probable *Albertosaurus* teeth within the underlying Drumheller Member extend the occurrence of this taxon to ~73 Ma [24, 42]. Tyrants Aisle is temporally correlated with the Drumheller Member, and it therefore seems probable that tyrannosaurid tracks from Tyrants Aisle (and elsewhere within Unit 4 of the Wapiti Formation) were produced by a species of *Albertosaurus* [35].

Theropod-like tracks below 45 cm in length are more challenging to identify, as a range of small-to-medium sized theropods with overlapping foot lengths are known from the Campanian–Maastrichtian of Laramidia, including members of Ornithomimidae, Dromaeosauridae, Troodontidae, Caenagnathidae, Alvarezsauridae, Therizinosauridae, and juvenile Tyrannosauridae [31, 54, 57, 105]. Furthermore, members of Thescelosauridae may theoretically produce theropod-like tracks up to approximately 30 cm in length [84]. Distinguishing these trackmaker types, where possible, relies predominantly on differing functional digit counts. For instance, tridactyl theropod-like tracks are unlikely to pertain to therizinosaurids or caenagnathids, which probably produced tetradactyl footprints [31, 108]. Di.Tw1.1.27D and Di.I.34E are attributable to deinonychosaurs on the basis of didactyly and, more specifically, to Troodontidae based on the relative shortness of digit IV in comparison to digit III [37]. Although we refrain from assigning most tridactyl theropod-like footprints below 45 cm in length to any particular clade, some of the larger and more distinct examples (e.g., Th.I.98-B, Th.I.8C, Th.I.89-A, and those within Th.Tw1.9B–6B) were possibly produced by ornithomimids based on their long, slender digits, occasional tendency for the impression of digit II to be separated from the rest of the print (present in Th.Tw1.2.8B), and constriction at the base of digit III (present in Th.I.89-A and Th.Tw1.4.6B) [85–87]. Rare, isolated ornithomimid bones are known from Unit 3 of the Wapiti Formation, and possibly Unit 4, but are generically indeterminate [8, 12, 14, 16]. Alternatively, some large tridactyl theropod tracks (such as Th.Tw1.4.6B) could pertain to juvenile tyrannosaurids, which are likely to have exhibited a similarly gracile foot morphology to that seen in ornithomimids [35].

### Possible trackmaker sociality

Aligned, unidirectional trackways may suggest the presence of multiple individuals moving side-by-side, providing evidence of gregarious behaviour [4, 7, 17]. However, such sequences can also be produced by a succession of solitary animals, at different times, which were ‘funnelled’ through the same space due to physical barriers, or were following the orientation of palaeoshorelines [109]. Therefore, it is important to consider additional evidence when postulating gregarious behaviour, such as trackway and print spacing, relative depth, and degree of preservational similarities [110].

Hadrosaurids—Closely associated and aligned large ornithopod trackways are a long-documented phenomenon known from Cretaceous tracksites around the world (e.g., [2, 3, 77, 111–115]). Skeletal and other evidence, including rare nesting sites (e.g., [116–118]) and common hadrosaurid-dominated bonebeds (e.g., [20, 25, 119–123]) further indicate widespread herding behaviour among these dinosaurs. More specifically, hadrosaurid-dominated bonebeds are also known from the Wapiti Formation [8, 55]. In this context, it is possible that the abundant hadrosaurid trackmakers at Tyrants Aisle were also engaging in gregarious behaviours.

**Fig 13:**
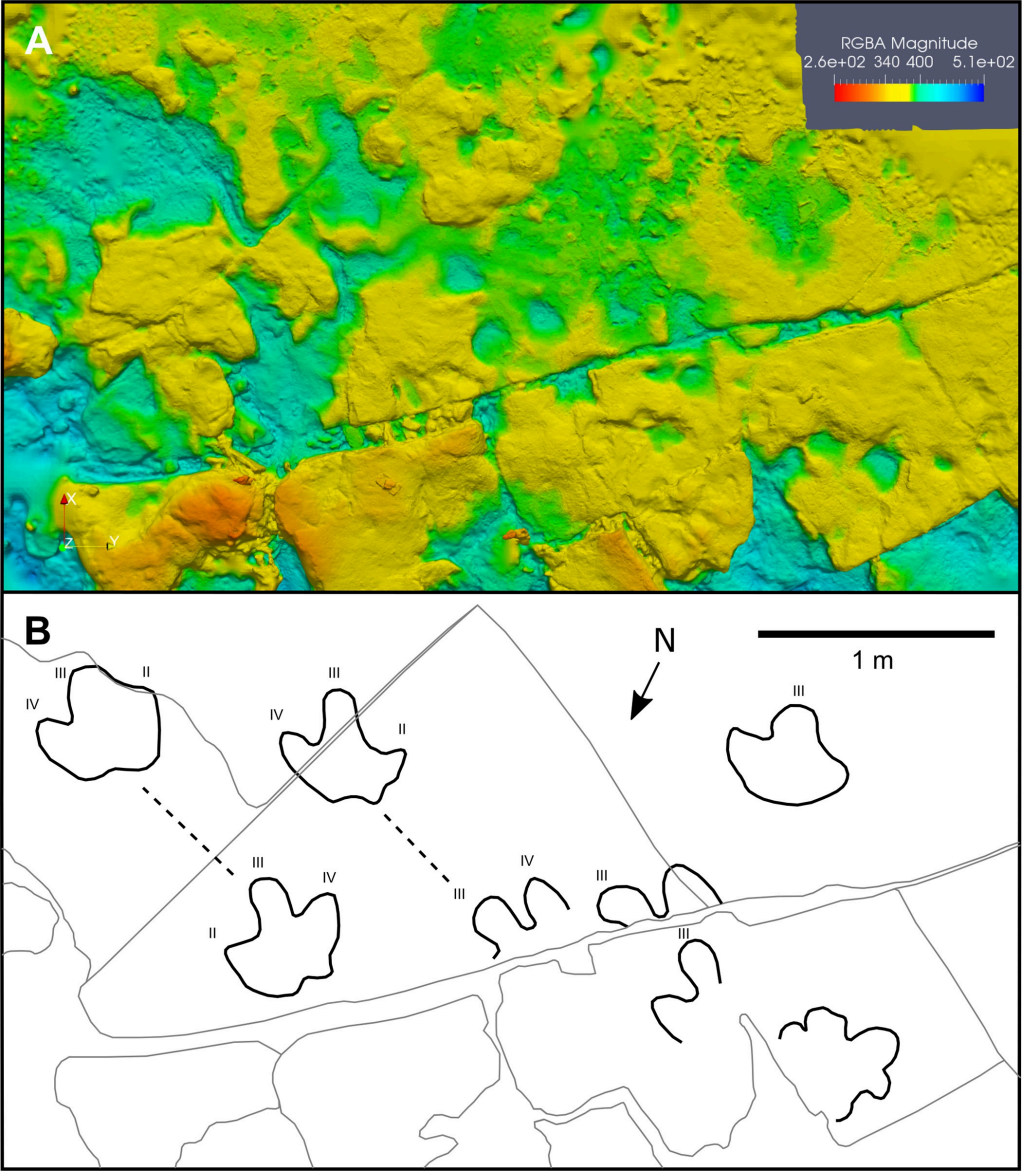
Aligned hadrosaurid footprints from track layer 1. **A**, digital elevation model, and **B**, outline drawing. The digital model in A—and the photographs used to create it—are available for download within the supplementary information.

The lowest track-bearing horizon (layer 1) at Tyrants Aisle preserves at least 15 hadrosaurid pes prints, some in single-step sequences (i.e., sets of exactly two successive tracks), and all concentrated within 15 linear metres of outcrop (Figs 3 and 13). No additional tracks have been observed in places where this same layer is exposed further west (Fig 1B). Nearly all of these hadrosaurid tracks, except H.I.28-B, were made by trackmakers travelling approximately SE (average = 143°). Due to the shortness and close proximity of the trackway sequences, it is difficult to confirm the precise number of individuals represented on this track layer. Nevertheless, between grid rows 28 and 33 on track layer 1, we estimate that five or more individuals were possibly walking side-by-side, based on the lateral spacing (~0.5–1.0 m apart) and non-overlapping arrangement of their tracks (Fig 13). By contrast, H.I.28-B is in opposition to the main trackway grouping and oriented in a westerly direction (Fig 3). H.I.28-B is also less eroded and more distinctly preserved than the surrounding tracks, and has an associated manus impression (Fig 8A–8C) that overprints the tip of digit III in H.I.28-C, which is part of the main grouping of aligned footprints. These attributes suggest H.I.28-B formed later than the aligned tracks. Thus, based on these trackway data, the concentration of prints within this relatively small area, and their relative degree of preservation, we conclude that the hadrosaurid tracks on layer 1 were produced during at least two temporally separated events: 1) a possible herd of at least 5 individuals walked across track layer 1 in a generally SE direction (Figs 3 and 13), and 2) at least one individual (the trackmaker of H.I.28-B) traversed the same area sometime later, travelling in a westerly direction (Figs 3 and 8A–8C). Evidence of possible hadrosaurid sociality is also present on track layer 3. In particular, two individuals may have been travelling NW together at a similar pace, separated by a lateral distance of about 1 m, between grid rows 52 and 54 (trackways identified as H.Tw6.53B–53A and H.Tw7.54A–54-A; Fig 1B; Table 2). However, it must be clarified that alternative scenarios, including the possibility that several lone individuals were ‘funnelled’ through the same area in succession, cannot be ruled out.

On both track layers 1 and 3, sets of aligned, regularly spaced hadrosaurid tracks are generally characterised by fairly uniform track lengths. If such sets of tracks document genuine social behaviour, the socialising individuals must have primarily been similar in size and, presumably, age (Figs 1B, 3 and 13; Table 1). Most other hadrosaurid tracks preserved across track layers 1 and 3 vary more widely in size and are largely isolated, providing no evidence of social behaviour. However, in one area of track layer 3 (Fig 10D and 10E), juvenile-sized and adult-sized tracks occur together within an area of 2 m^2^; H.I.71A is the smallest confidently identified hadrosaurid track at Tyrants Aisle (track length = 23 cm), and occurs less than one metre from H.I.72A (track length = 33cm), both of which are similarly oriented. Two larger hadrosaurid footprints (track length≈53 cm) are preserved close to H.I.71A and H.I.72A (Fig 10D and 10E), but are only roughly comparable to H.I.71A and H.I.72A in orientation. Therefore, it is uncertain whether the hadrosaurids at Tyrants Aisle were consistently or only occasionally age segregated, if herding was indeed taking place.

The occasional presence of manus impressions alongside pes prints (e.g., in H.I.-2B, H.Tw1.2.2A, H.I.28-B, and H.I.72A) indicates that some of the hadrosaurids that left traces on track layers 1 and 3 were walking quadrupedally. By contrast, the majority of the hadrosaurid pes tracks lack associated manus impressions. As most of the animal’s body weight was supported by the pedes—indicated by pronounced heteropody and a centre of mass inferred to have been positioned dorsal to the pedes [124–126]—smaller manus impressions may have failed to register on the substrate or were shallowly impressed, and thus more easily lost by erosion, which is prevalent at Tyrants Aisle. Despite this, we acknowledge the possibility that in some cases the absence of manus impressions may reflect a genuinely bipedal mode of walking.

Probable theropods—The parallel alignment of two particular trackways on track layer 3 (Th.Tw4.71A–70-A and Th.Tw5.71-A–72-A) may suggest social behaviour between theropods (hip height≈0.72 m) at Tyrants Aisle (Fig 10). Both animals were walking in a roughly NW direction, ~1 m apart, with similar pace and stride lengths (Table 2). A possible trackway from a third individual was identified, and is oriented in the same direction, between the two main trackways (Fig 10). However, this possible sequence is heavily eroded and difficult to interpret with confidence. Quality of track preservation varies between and within the two main trackways. Print Th.Tw5.3.72-A is relatively clear, whereas the preceding two footprints in the same trackway are more poorly defined. In the adjacent trackway, Th.Tw4.4.70-A retains only the impressions of the distal ends of the digits, while Th.Tw4.1.71A is represented by a single curved trace of digit III. Furthermore, track Th.Tw4.2.70A is entirely mud collapsed, exhibiting only thin creases pertaining to each digit (Fig 10D and 10E). By contrast, the three tracks within Th.Tw5.71-A–72-A, although variable in morphology and preservational clarity, are not similarly mud collapsed. These differences may suggest the two individuals traversed track layer 3 at different times. However, given that preservation is also highly variable within each trackway, we suggest that the differences in preservation between Th.Tw4.71A–70-A and Th.Tw5.71-A–72-A are instead due to differential sediment properties and erosion. Based on the close association between these two trackways, their similar pace lengths, and the observation that small tridactyl theropod-like tracks are rare elsewhere across the ~1, 400 m^2^ tracksite (n≈4, not including these trackways), we conclude that the two trackmakers of Th.Tw4.71A–70-A and Th.Tw5.71-A–72-A were probably travelling together. These data are consistent with trackway (e.g., [17, 127–131]) and skeletal (e.g., [132–136]) evidence from elsewhere that indicates gregariousness—whether periodic or sustained—was present in a range of theropod clades and body sizes.

### Palaeoecological insights

**Fig 14:**
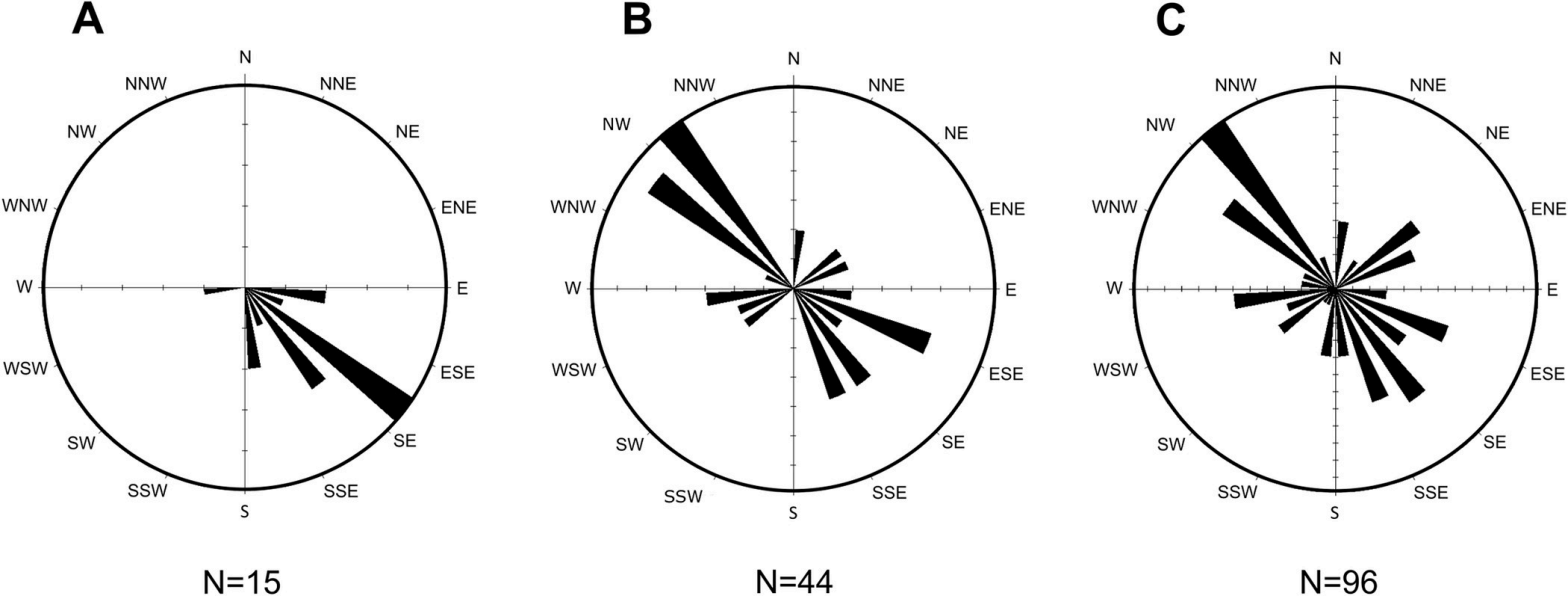
Rose diagrams of *in-situ* track orientations based on digit III azimuths. **A**, Track layer 1 (confident traces only), **B**, track layer 3 (confident traces only), and **C**, track layer 3 (all measurable traces). Number of traces contributing to each plot as indicated.

Based on the footprint orientation data, the majority of trackmakers on layer 1 were walking in a SE direction (Fig 14A), whereas those on layer 3 show a more bi-directional pattern aligned along the SE–NW axis (Fig 14B and 14C). Although the average direction of current flow during sediment deposition was northward (see ‘depositional environment, tracksite stratigraphy and lithology’), some *Rhizocorallium* on both track layers 1 and 3 are similarly oriented SE–NW. Therefore, some of the trackmakers were likely following the orientation of a meandering river margin.

**Fig 15:**
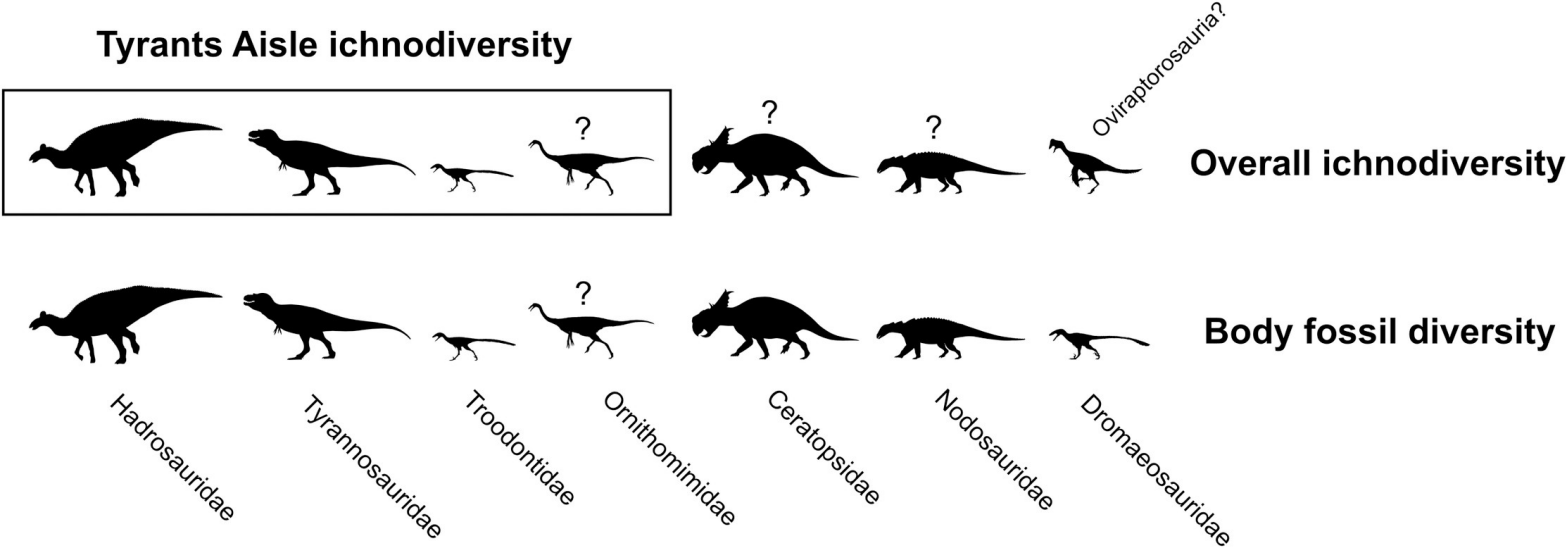
Non-avian dinosaur diversity from Tyrants Aisle compared with the collective fossil record of Unit 4 of the Wapiti Formation. Taxonomic resolution is limited to “family” level or higher. Question marks indicate possible presence. Taxonomic presence from the skeletal record is based on [8, 10, 14–16], while occurrences from the track record are collated from [8, 10, 11, 17, 35–37] and herein. Silhouettes are not to scale and were sourced from PhyloPic. Each silhouette is available under CC-BY license, with credit given to Nobu Tamura, T. Michael Keesey, Tasman Dixon, Scott Hartman, Andrew A. Farke, and Jaime Headden.

Dinosaurian ichnodiversity at Tyrants Aisle is a subset of that captured within the entire track and body fossil record of Unit 4 of the Wapiti Formation (Fig 15). The largest and most abundant tracks in the Tyrants Aisle assemblage are those of hadrosaurids, the only ornithischian trackmakers identified at the site. Hadrosaurid prints occur on both of the main track layers 1 (number of definitive pes tracks ≥15; manus tracks ≥2) and 3 (number of definitive pes tracks ≥25; manus tracks ≥4). On track layer 1, only hadrosaurid tracks are present. On track layer 3, an abundance ratio of 25:1:23 is obtained for all confidently identifiable hadrosaurid, tyrannosaurid, and non-tyrannosaurid theropod-like pes tracks, respectively. When trackways are treated as a single occurrence, the faunal ratio for track layer 3 becomes 17:1:14. However, these ratios do not include the high amount (≥90) of heavily eroded and non-diagnostic tracks on layer 3, which could not be reliably counted. Many indeterminate tracks on layer 3 appear as subrounded, eroded potholes, whose large size indicates that they likely represent former hadrosaurid tracks. Therefore, the true proportion of hadrosaurid trackmakers is probably even higher than these ratios imply. Furthermore, as tyrannosaurid tracks below 45 cm in length were not readily distinguishable from other tridactyl theropod-like track types, the number of tyrannosaurid tracks recognised here is likely also an underestimate. Nevertheless, the available data across all track layers collectively support an overall palaeoecological dominance of hadrosaurids at Tyrants Aisle, corroborating their skeletal abundance both within Unit 4 of the Wapiti Formation [8, 15, 16], and broadly across many Late Cretaceous terrestrial ecosystems within Laramidia [24, 54, 57, 137–139]. Similarly, a relative scarcity of tracks attributable to probable troodontids [37] and possible ornithomimids is congruent with their sparse body fossil record in the Wapiti Formation. Except for Th.I.98-B and Ty.I.OG, all of the theropod-like tracks at Tyrants Aisle occur on track layer 3, although this is likely due to the much greater exposure of this particular layer (Fig 1B).

Confidently measurable hadrosaurid tracks at Tyrants Aisle range from ~23–65 cm in length, with most tracks measuring between 45 and 65 cm long (Table 1), suggesting that the majority of individuals were between ~1.8 m and ~2.6 m tall at the hip. By comparison, ROM 801—one of the heaviest known individuals of *Edmontosaurus regalis—*stood approximately 3 metres tall at the hip and has a reconstructed pes ~66.5 cm in length [140, 141]. Among hadrosaurids, Horner et al. [142] and Evans [143] consider juveniles to be individuals measuring 50% or less than the maximum known adult body length. Thus, as most hadrosaurid footprints at Tyrants Aisle approach the predicted pes length of ROM 801, the majority of hadrosaurid trackmakers at Tyrants Aisle were likely adults. Track assemblages such as this, in addition to relative skeletal rarity within the body fossil record, suggest that large *Edmontosaurus* individuals exceeding 3.0 metres tall at the hip constituted only a small minority of a given population [144].

Ceratopsids—whose tracks usually possess five manual and four pedal digits [4]—are conspicuously undocumented from the Tyrants Aisle track fauna, but are known from body fossils within Unit 4 of the Wapiti Formation [14]. Specifically, the centrosaurine *Pachyrhinosaurus* is abundant within two distinct monodominant bonebeds, which are situated on Pipestone Creek near the Unit 3–Unit 4 boundary [13, 14], and on the Wapiti River near the middle of Unit 4 [14]. Thus, Tyrants Aisle is stratigraphically positioned between these two bonebeds and falls within the stratigraphic range of *Pachyrhinosaurus* within the Wapiti Formation. We attribute the lack of identifiable ceratopsid tracks to inadequate sampling, as many footprints at Tyrants Aisle are heavily eroded and could not be identified. Alternatively, their absence may simply reflect a chance mismatch between *Pachyrhinosaurus* herd movements and track layer deposition.

### Context within the Laramidian vertebrate fossil record

**Fig 16:**
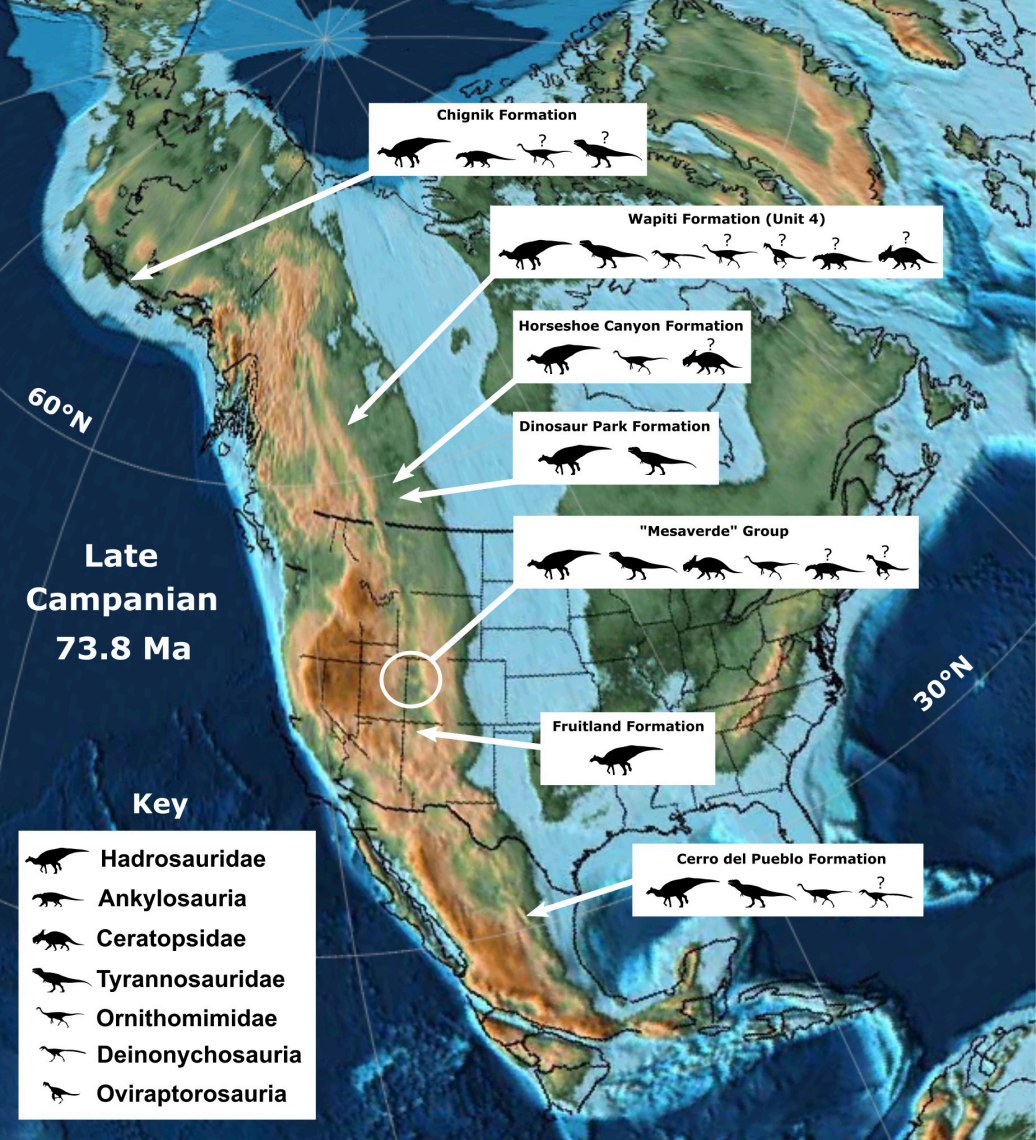
Palaeogeographic map of North America during the late Campanian (~73.8 Ma), adapted from map 18 of the Scotese [157] PALEOMAP PaleoAtlas for GPlates, showing the distribution and ichnodiversity of major dinosaur track-bearing units from the late Campanian of Laramidia. Strata of each unit are not necessarily contemporaneous with one another. The first silhouette in each box represents the most abundant trackmaker type in that unit. Question marks indicate uncertain trackmaker occurrences. Silhouettes are not to scale and were sourced from PhyloPic. Each silhouette is available under CC-BY licence, with credit given to Nobu Tamura, T. Michael Keesey, Tasman Dixon, Scott Hartman, Andrew A. Farke, and Jaime Headden.

Inclusion of Tyrants Aisle within a synthesis of late Campanian dinosaur ichnofaunas from Laramidia reinforces a near-universal abundance of hadrosaurid trackmakers (Fig 16). Alaskan ichnoassemblages within the Chignik and lower Cantwell formations are most often dominated by *Hadrosauropodus*, although the latter unit is probably early Maastrichtian in age [29, 31, 32]. Similarly, late Campanian track horizons within the Oldman, Dinosaur Park, Horseshoe Canyon, and St. Mary River formations of southern Alberta, as well as the ‘Mesaverde Group’ of Utah, Wyoming, Colorado, and the Fruitland Formation of New Mexico, are all hadrosaurid-dominated [3, 43, 60, 75, 87, 145–153 and references therein] (Fig 16). In Mexico, ichnofaunas of the Cerro del Pueblo Formation are hadrosaurid-dominated in terms of footprint abundance, although theropod tracks occur at a larger number of tracksites [139, 154–156].

**Fig 17:**
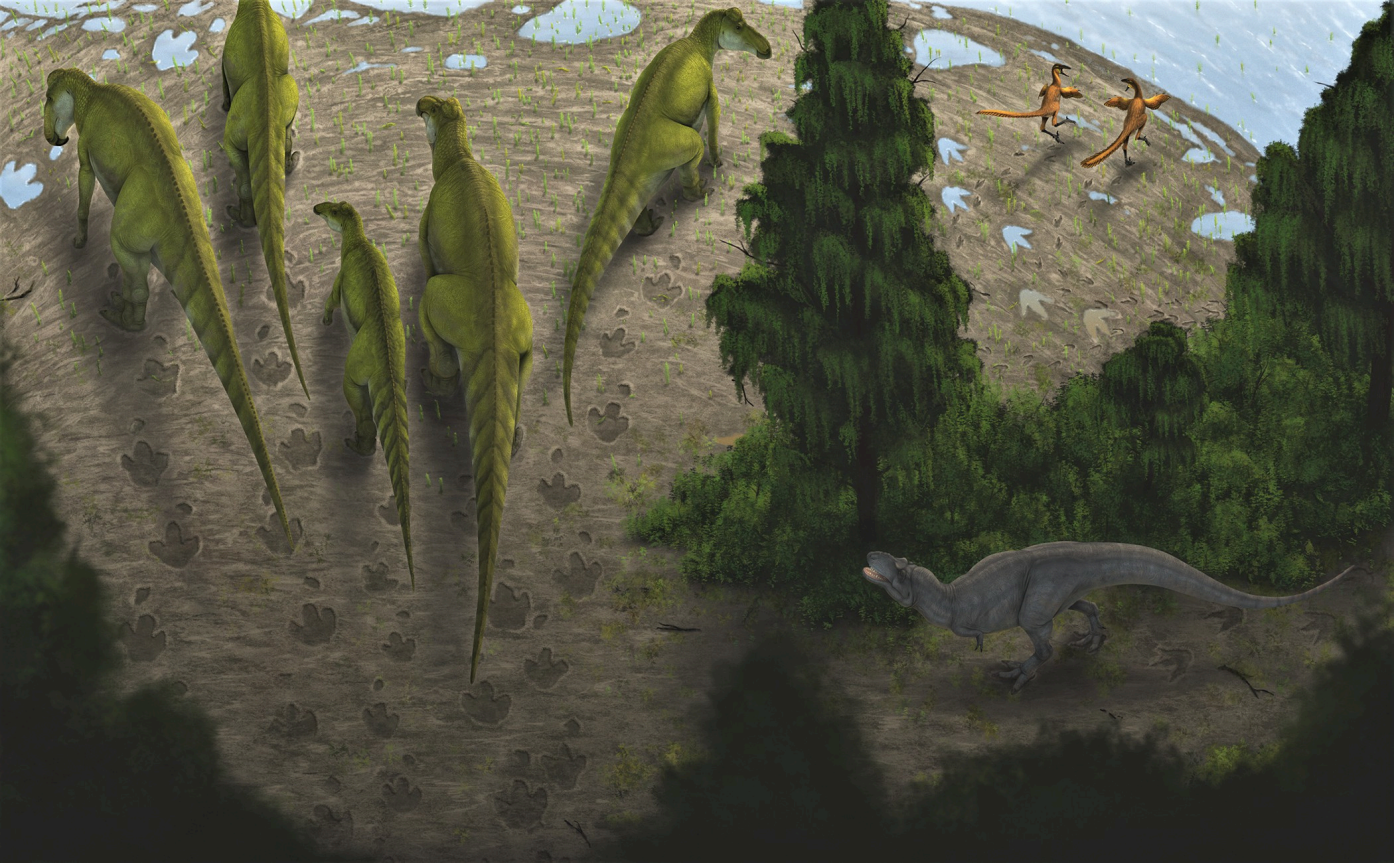
Hypothetical reconstruction of the Tyrants Aisle tracksite during track formation, depicting a small herd of *Edmontosaurus regalis* as they produce tracks in a riverine setting. A lone tyrannosaurid watches the herd, while a pair of troodontids scuffle nearby. Artwork by José Vitor Silva.

Tracks of ceratopsids, ankylosaurians, caenagnathids, and deinonychosaurs are the least frequently observed late Campanian dinosaur ichnites within Laramidia, with no distinct pattern in their latitudinal occurrence, especially once skeletal records are also considered [54, 57, 158] (Fig 16). Ceratopsids are known to dominate some ichnofaunas, such as those of the Laramie Formation in Colorado, although these horizons are Maastrichtian in age [159]. Tyrants Aisle, and the fossil record of the Wapiti Formation in general, is therefore in palaeoecological agreement with other ichnofaunas that collectively demonstrate the critical role of hadrosaurids as primary megaherbivores across Laramidia during the late Campanian, allowing for more faithful palaeocommunity reconstructions (Fig 17). Additional studies should further assess the role of lithology and palaeoenvironment as a determinant of observed dinosaur ichnodiversity—across a broad sample of tracksites—as this may further improve understanding of Laramidian dinosaur palaeoecology by clarifying the influence of depositional setting on the faunal signals identified herein.

## Supporting information

S1 Appendix Photogrammetric model of hadrosaurid tracks H.I.28-B and H.I.28-C on track layer 1. For scale, the blue writing board measures 11.8 cm wide and 18.7 cm tall.
(PLY)

S2 Appendix Photogrammetric model of *ex-situ* hadrosaurid track natural cast H.I.3I. For scale, H.I.3I measures 64 cm in length.
(PLY)

S3 Appendix Photogrammetric model of a portion of track layer 3 containing the tyrannosaurid track Ty.I.8C, cf. *Ornithomimipus* trackway Th.Tw1.9B–6B, and other tridactyl theropod-like and hadrosaurid tracks. See Fig 4 of [35] for a diagrammatic illustration of the same area. For scale, the blue writing board measures 11.8 cm wide and 18.7 cm tall.
(PLY)

S4 Appendix Photogrammetric model of cf. *Ornithomimipus* track Th.Tw1.4.6B and indeterminate tridactyl track Tri.I.5B on track layer 3. For scale, the blue writing board measures 11.8 cm wide and 18.7 cm tall.
(PLY)

S5 Appendix Photogrammetric model of large theropod track Th.I.98-B on track layer 2. The scale card in the model measures 9.5 cm in length.
(PLY)

S6 Appendix Photogrammetric model of aligned, probable small theropod trackways Th.Tw4.71A–70-A and Th.Tw5.71-A–72-A on track layer 3, with several hadrosaurid tracks nearby. For scale, the blue writing board measures 11.8 cm wide and 18.7 cm tall.
(PLY)

S7 Appendix Photogrammetric model of probable theropod track Th.Tw3.1.27D and probable troodontid tracks Di.Tw1.1.27D and Di.Tw1.2.27D on track layer 3. For scale, the area shown in the model measures 109 x 100 cm.
(PLY)

S8 Appendix Photogrammetric model of indeterminate morphotype A trackway In.Tw1.76-A–77-A and hadrosaurid track H.I.76-B on track layer 3. For scale, the blue writing board measures 11.8 cm wide and 18.7 cm tall.
(PLY)

S9 Appendix Photogrammetric model of indeterminate morphotype B track Tri.I.4B on track layer 3. For scale, Tri.I.4B measures 37.5 cm in track width.
(PLY)

S10 Appendix Photogrammetric model of indeterminate morphotype B track Tri.I.10C and probable small theropod track Th.I.10C on track layer 3. For scale, the blue writing board measures 11.8 cm wide and 18.7 cm tall.
(PLY)

S11 Appendix Photogrammetric model of several aligned hadrosaurid tracks on track layer 1. For scale, the area shown in the model measures 5.45 m in greatest width.
(PLY)
