# Correction: The dinosaur tracks of Tyrants Aisle: An Upper Cretaceous ichnofauna from Unit 4 of the Wapiti Formation (upper Campanian), Alberta, Canada

**DOI:** 10.1371/journal.pone.0319221

**Published:** 2025-02-07

**Authors:** The PLOS ONE Staff

Table 2 is formatted incorrectly. Please see the correct Table 2 here.

**Table 2:** Trackway parameters for identified hadrosaurid and probable theropod sequences.

Trackway number	Trackway type	N	L/R	CBT	PL	SL	PA	FR	Hip height	Speed (m/s)
H.Tw1.-2B–2A	Hadrosaurid	3 (one missing)	L: (4?) R: (1, 3?)	270°?*	1.29*: (3–4)	2.44?: (1–3)	-	-	2.36 based on (1)	1.27?
H.Tw2.14B–12E	Hadrosaurid	3	L: (1, 3) R: (2)	122°*	2.01*: (1–2) 1.77*: (2–3)	3.72*: (1–3)	173.5°*	-3.5°*: (2)	2.40 based on (2)	2.52
H.Tw3.30-C–29-B	Hadrosaurid	2	L: (2) R: (1)	130°*	1.22*	-	-	0°*: (1) -9°*: (2)	2.02 based on (1)	-
H.Tw4.31-C–31-B	Hadrosaurid	2	L: (2) R: (1)	130°*	1.13*	-	-	-20°*: (1) -19*: (2)	1.98 based on (2)	-
H.Tw5.38B–31H	Hadrosaurid	1–5?	-	097°?*	-	-	-	-9°?*: (2)	2.70 based on (2)	-
H.Tw6.53B–53A	Hadrosaurid	2	L: (1) R: (2)	297°*	1.10*	-	-	-33°*: (1) -5°*: (2)	2.48 based on (2)	-
H.Tw7.54A–54-A	Hadrosaurid	2	L: (2?) R: (1?)	319°?*	0.96*	-	-	+5°?*: (1) +3°?*: (2)	2.38 based on (1)	-
H?.Tw9.69J–71G	Hadrosaurid?	1–4?	-	297°?*	1.17*: (1–2) 1.46*: (2–3) 1.08*: (3–4)	2.64*: (1–3) 2.54*: (2–4)	175°*: (1–3) 173.5°*: (2–4)	-	-	-
H?.Tw10.71H–74J?	Hadrosaurid?	1–4?	-	-	1.23*, 1.46*, 1.11* (order unknown)	2.66*, 2.55* (order unknown)	-	-	-	-
Th.Tw1.9B–6B	Ornithomimid or juvenile tyrannosaurid	3 (one missing)	L: (2, 4) R: (1)	056°*	1.27*: (1–2)	2.58*: (2–4)	-	-4°*: (1) -13°*: (2) -2°*: (4)	1.62 based on (4)	2.17
Th.Tw2.9B–9C	Indet. small theropod-like	2	L: (1) R: (2)	109°*	0.89*	-	-	-16°*: (1) +19°*: (2)	0.72 based on (2)	-
Th.Tw3.27D–24H	Indet. medium theropod-like	3–4?	L: (2, 4) R: (1)	~110°*	1.67*: (1–2)	3.55*: (2–4)	-	+15°*: (1) -1°*: (2) +10°*: (4)	1.16 based on (1, 4)	5.46
Th.Tw4.71A–70-A	Indet. small theropod-like	4	L: (1, 3) R: (2, 4)	330°	0.47*: (1–2) 0.36: (2–3) 0.49: (3–4)	0.76: (1–3) 0.84: (2–4)	132°*: (1–3) 149°*: (2–4)	+4°*: (2) -9°*: (3) -6°*: (4)	-	-
Th.Tw5.71-A–72-A	Indet. small theropod-like	3	L: (2) R: (1, 3)	326°	0.49: (1–2) 0.54: (2–3)	0.86	113°*	+18°*: (1) -17°*: (2) +4°*: (3)	0.72 based on (3)	0.89
Di.Tw1.27D–29D	Probable troodontid	1–4? (one or more missing?)	L: (1, 3?) R: (2?)	~257°*	-	-	-	-	0.48 based on (1)	-

Abbreviations: N = number of prints preserved within trackway, with any missing tracks indicated in brackets; L = left foot; R = right foot; CBT = 360° compass bearing of the trackway midline (east from north); PL = pace length; SL = stride length; PA = pace angulation; FR = foot rotation, where (-) indicates inward rotation, and (+) indicates outward rotation. Data for L, R, PL, SL, PA, FR, and hip height are accompanied by bracketed numbers, which identify specific tracks within a particular trackway e.g., (3) identifies the third track within the trackway. Linear measurements are in metres. Asterisks (*) denote measurements taken digitally in Inkscape v. 0.92 from either a scaled photograph, map, or a still taken from a digital elevation model. Question marks indicate uncertain measurements, due to ambiguous print association, or cases where the foot which produced one or more tracks (i.e., left or right) is not certain. See Fig 5C and 5D for a visual demonstration of trackway measurements.

The publisher apologizes for the error.
